# Heavy Metal Pollution in Arid Urban Environments: Anthropogenic and Geogenic Insights from Road Dust in the United Arab Emirates

**DOI:** 10.1007/s00244-025-01145-6

**Published:** 2025-08-29

**Authors:** Yousef Nazzal, Alina Bărbulescu, Manish Sharma, Fares Howari, Imen Ben Salem, Rania Dghaim, Pramod Kumbhar, Cijo M. Xavier, Suhail Alghafli, Ahmed A. Al-Taani, Mutaz Mohammad, Azzah Nasser Salem Nayem Alkaabi, Saif Nazzal, Cristian Ștefan Dumitriu

**Affiliations:** 1 College of Natural and Health Sciences, Zayed University, P.O. 144534, Abu Dhabi, United Arab Emirates; 2 Department of Civil Engineering, Transilvania University of Brașov, 5 Turnului Str., 500152 Brașov, Romania; 3 School of Earth, Atmosphere & Environment, Monash University, Melbourne, Australia; 4 College of Humanities and Sciences, Ajman University, P.O. 346, Ajman, United Arab Emirates; 5 SAgE Technical Services, Newcastle University, Newcastle Upon Tyne, UK; 6 Department of Earth and Environmental Sciences, Yarmouk University, Irbid, Jordan; 7 York University, Toronto, ON Canada; 8 Faculty of Mechanical Engineering and Robotics in Construction, Technical University of Civil Engineering, Calea Plevnei 59, 010234 Bucharest, Romania

## Abstract

Dust is a significant environmental concern due to its pervasive nature and potential health risks, particularly from heavy metals. This is exacerbated in urban areas, where dust can act as a reservoir for pollutants, posing risks to human health through various exposure pathways. This study aims to explore and compare the distribution of heavy metals in road dust from two distinct cities in the UAE: Dubai, a commercial hub, and Khor Fakkan, a coastal town with industrial activities. Road dust samples were collected from 29 locations in both cities, including areas with varying traffic density, residential settings, and industrial zones. Findings reveal notable levels of Cd exceeding background levels across both regions, with a slightly higher range (18.05–47.99 mg/kg) in Khor Fakkan compared to (13.96–44.03 mg/kg) in Dubai. Similarly, Zn levels peak at 587.88 mg/kg in certain samples in Dubai and reach 1802.02 mg/kg in Khor Fakkan. Principal Component Analysis highlights Co, Fe, Cd, and Zn as primary pollutants in Dubai, while Ni, Cr, Cu, and Co are predominant in Khor Fakkan. Overall, pollution index analyses, including the geoaccumulation index, pollution index, and enrichment factor, underscore Cd, Zn, and Ni as key pollutants across both regions, with hotspots associated with industrial and vehicular emissions. Representative series of the EFs are also presented, emphasizing the average extent of pollution with various heavy metals. Future work should focus on source attribution analyses and risk mitigation strategies to reduce heavy metal pollution in urban environments and protect public health and ecosystems.

**Graphical abstract:**

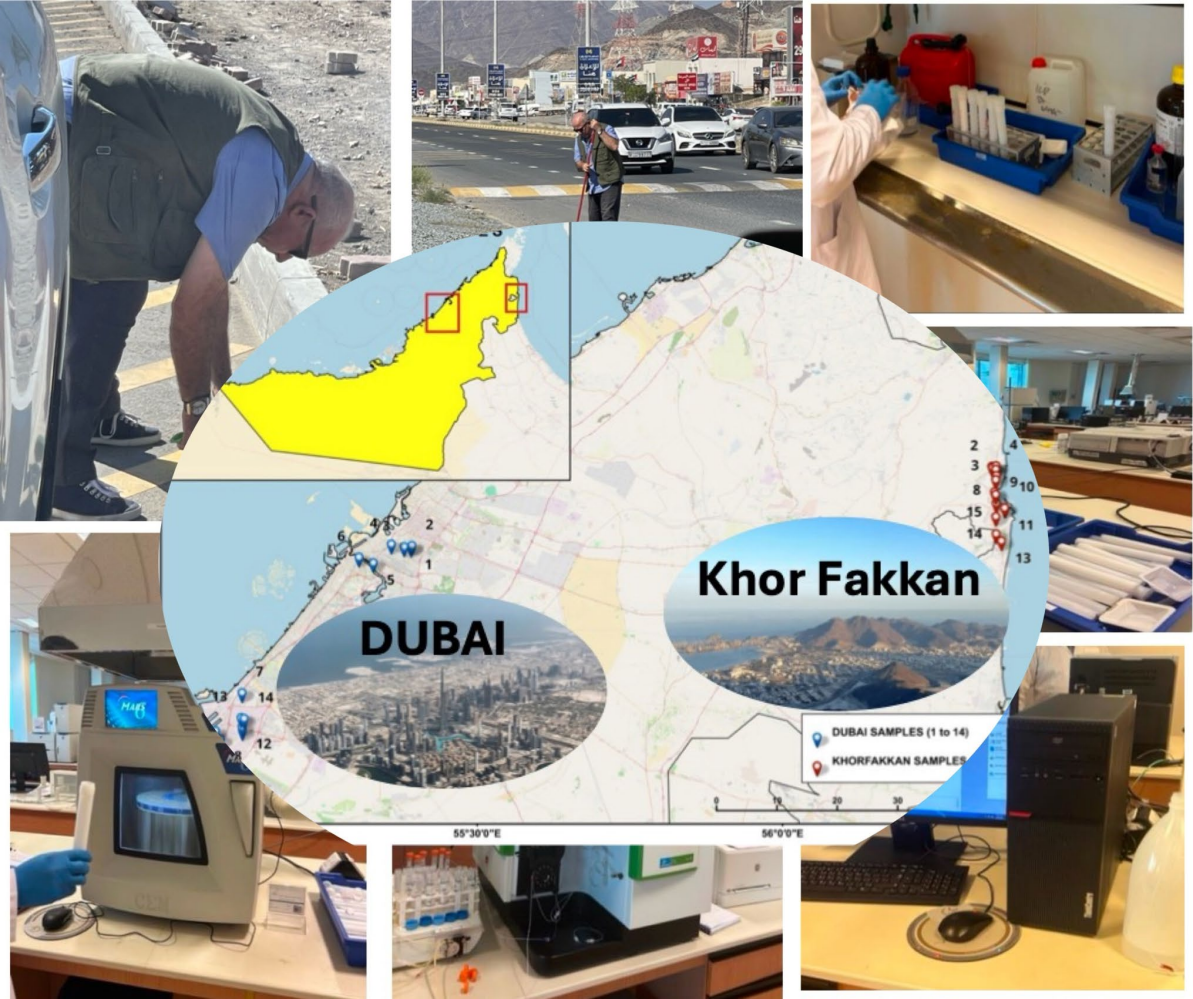

Among the three terrestrial materials—soil, sediment, and dust—originating from the Earth’s crust, dust is the most pervasive and poses the most significant risk to human health (Yongming et al. 2006). Its heterogeneous composition, which includes both organic and inorganic fractions, combined with multiple exposure pathways such as inhalation, ingestion, and skin contact, makes dust a significant health concern for both children and adults (Khodadadi et al. 2022; Faisal et al. 2021).

Several studies have indicated urban road dust as a temporary reservoir for heavy metals from local and regional sources. Due to its ability to suspend high concentrations of pollutants like heavy metals, polycyclic aromatic hydrocarbons (PAHs), and polychlorinated biphenyls (PCBs), it can act as a source of these contaminants, which, under certain atmospheric conditions, can be resuspended in the air, contributing to atmospheric pollution (Roy et al. 2022; Amato et al. 2010; Moreno et al. 2013; Lu et al. 2009; Manno et al. 2006; Bilos et al. 2001).

Road dust characterization is essential for assessing urban environmental pollution and associated health risks to the population (Yongming et al. 2006). Research indicates that fine particulate matter with a diameter of less than 2.5 μm (PM2.5) can penetrate the alveoli and cause significant harm to human health. Brown et al. (1950) reported that particles sized 5–10 μm are retained in the nasal chamber, with retention decreasing as particle size decreases, whereas particles smaller than 1 μm (PM1.0) are retained beyond the nasal chamber in the lower respiratory tract, including the trachea-bronchial and alveolar regions. These particles, with an aerodynamic size of less than 1 μm, contain PCBs, PAHs, toxic heavy metals, and mineral trace elements from sources such as tire and brake wear tear, exhaust from vehicles, traffic, solid waste burning, industries, and other combustion sources in urban areas (Lu et al. 2009).

The potential risks of inhaling and ingesting heavy metals in urban areas are a major concern due to their association with various health issues, including respiratory and cardiovascular diseases, neurological disorders, and cancer (Inyang and Bae 2006). Heavy metal toxicity tends to bioaccumulate in human tissues, increasing the concentration of heavy metals within a biological organism over time when exposed to a particular environment (Du et al. 2013). Even at low concentrations, many heavy metals are toxic and have carcinogenic effects (Willers et al. 2005; Zhang et al. 2012). Their bioaccumulation in the human body can also induce oxidative stress due to the production of reactive oxygen species (ROS) (Coman and Draghici 2011). Furthermore, combined intake of different heavy metals can result in toxicity that may be antagonistic, additive, or synergistic (Tchounwou et al. 2012; Wang and Fowler 2008). Coman and Draghici (2011) have demonstrated that the combined effects of arsenic (As), cadmium (Cd), and lead (Pb) caused severe renal dysfunction, which was more pronounced than the effects of each metal.

The growing concern about roadside dust’s impact on public health is global. However, there is still a need for more localized studies to understand the specific sources, composition, and health effects in different regions. With rapid urbanization, increasing vehicular traffic, and an arid climate characterized by frequent dust storms, understanding the composition and health impacts of roadside dust in the United Arab Emirates (UAE) is vital for effective environmental and public health management (Al-Taani et al. 2021; Nazzal et al. 2021a, 2021b; Maloukh et al. 2023). Among the various contributors to airborne particulate matter in the UAE, non-exhaust vehicle emissions—particularly from tire and brake wear—have emerged as a significant yet often underrecognized source of environmental pollution. The UAE’s rapid economic growth has led to a dramatic increase in vehicle ownership, with over 3.5 million registered vehicles by 2020, including 1.8 million in Dubai alone (RTA Dubai 2020). This expansion has created notable environmental challenges due to non-exhaust emissions, which are frequently overlooked compared to traditional exhaust pollutants. Tires are composed of natural rubber, synthetic rubber (especially styrene-butadiene), carbon black, silica, steel cords, and various chemical additives tailored for the UAE’s extreme climate—marked by high heat, friction, and intense UV exposure (Mayer et al. 2024). Brake systems are made of composite materials including metallic particles like copper, steel, and iron, as well as phenolic resin binders and friction modifiers (Grigoratos and Martini 2015). These components release fine particles and microplastics during normal vehicle operation, with brake pads generating particulates through friction and tires degrading into microplastic fragments (Panko et al. 2013; Thorpe & Harrison 2008). The UAE’s dry and windy conditions exacerbate this issue by promoting the suspension and persistence of these particles in the air. Habib et al. (2022) & Abbasi et al. (2017) confirmed the presence of synthetic rubber polymers and metallic particles in urban dust samples across the UAE and adjacent countries, directly linking these emissions to environmental contamination. Research indicates that such non-exhaust sources significantly contribute to urban air pollution, depositing persistent microplastics and heavy metals into air, soil, and possibly groundwater systems.

The UAE has seen a significant expansion in its road network—from 4080 km in 2008 to over 18,255 km by 2020—which has intensified population exposure to road dust, particularly among those living near major roads and highways (Al-Taani et al. 2019a; Nazzal et al. 2021b). Elevated metal concentration in dust have been reported from vehicular emissions, industrial activities, and dust from the Arabian Gulf, a region with extensive oil and gas reserves contributing to significant atmospheric metal emissions (Cai and Li 2019; Al-Taani et al. 2023; Nazzal et al. 2023; Batayneh et al. 2014). Semerjian et al. (2024) have reported iron (Fe), manganese (Mn), chromium (Cr), and nickel (Ni) as primary contaminants, attributed mainly to heavy traffic in Sharjah’s commercial and industrial zones. Indoor dust in Dubai has shown enrichment factors for calcium (Ca), copper (Cu), iron (Fe), manganese (Mn) linked to local soil lithology and industrial activities, with dust transported by storms (Nazzal et al. 2023). Maloukh et al. (2023) also suggested that bacterial communities in UAE outdoor dust are not related to infectious agents but are adapted to the arid climate, aiding in pathogen surveillance and public health decision-making.

Due to climate change and rapid urbanization, predicting the complex dynamics of long-range atmospheric dust transport and short-range interception has become increasingly challenging (Eqani et al. 2016). Inhabitants are frequently exposed to heavy metals through street dust.

Despite the growing body of literature on heavy metal contamination in road dust, there remains a significant gap in localized studies focusing on arid and semi-arid regions, particularly in the Arabian Peninsula. The United Arab Emirates (UAE), with its rapid urbanization, diverse geology, and frequent dust events, presents a critical yet under-investigated case for understanding the dynamics of dust-bound metal pollutants and their potential public health implications.

This study addresses this gap by pursuing three key objectives: (1) to conduct a comparative assessment of heavy metal contamination in road dust from two geologically and ecologically distinct UAE cities, urban-coastal Dubai and mountainous-coastal Khor Fakkan; (2) to apply a comprehensive suite of pollution assessment tools, including individual indices (I_geo, PI, EF, CF) and composite indices (PLI, CPI, CD, mCD), to quantify and contextualize contamination levels; and (3) to employ advanced analytical approaches, such as EF_local enrichment modeling, k-medoids clustering, and Principal Component Analysis (PCA), to differentiate natural and anthropogenic metal sources and visualize spatial contamination patterns at both city-wide and neighborhood scales. By integrating these diverse methodologies, this study provides a robust diagnostic framework for assessing urban environmental risks in arid environments, a novel approach in the regional context. The findings aim to support future environmental monitoring programs, inform regulatory action, and guide public health interventions in the UAE and other rapidly developing arid regions globally.

## Study Area and Experiments

### Study Area

Dubai, a commercial hub, is renowned for its futuristic skyline, luxurious lifestyle, and ambitious architectural projects. In contrast, Khor Fakkan, a coastal town, is known for its stunning beaches, crystal-clear waters, scenic Hajar Mountains, and significant industrial activities, including port operations and heavy vehicular traffic. Both cities, located in the UAE, exhibit distinct geological settings that influence local environmental conditions and the distribution of heavy metals in road dust.

Dubai lies on flat coastal plains dominated by Quaternary alluvial and aeolian deposits, shaped largely by wind and fluvial processes (Al-Taani et al. 2019b). This geomorphology, combined with intense urbanization and industrial emissions, contributes to the accumulation of metals such as lead (Pb) and cadmium (Cd) in urban dust (Cai and Li 2019). Khor Fakkan, positioned along the Gulf of Oman, borders the rugged Hajar Mountains and is underlain by ophiolitic rocks like peridotites and gabbros from the Semail Ophiolite (Joun et al. 2019). These ultramafic formations naturally contain nickel (Ni) and chromium (Cr), contributing to elevated levels of these metals in local dust.

Although both cities are urbanized, Khor Fakkan’s mountainous terrain introduces distinct topographical and lithological influences. Its classification as ‘mountainous’ reflects this geologic context rather than a rural character. Elevated concentrations of Ni and Cr in the area may result from natural weathering of surrounding ophiolitic rocks. However, industrial activities and port operations appear to be the dominant sources of contamination, as shown by extremely high concentrations at industrial sampling sites (Al-Taani et al. 2019b; Arslan 2001). This setting highlights the complex interplay between natural and anthropogenic sources in shaping heavy metal distribution in road dust.

Both cities experience similar climatic conditions, with average annual temperatures around 27–28 °C, extremely hot summers often exceeding 40 °C, and mild winters (National Centre of Meteorology-UAE). Annual precipitation averages 100 mm in Dubai and 150 mm in Khor Fakkan, further influencing dust dynamics and environmental behavior of heavy metals.

### Sampling and Laboratory Analysis

**Fig. 1:**
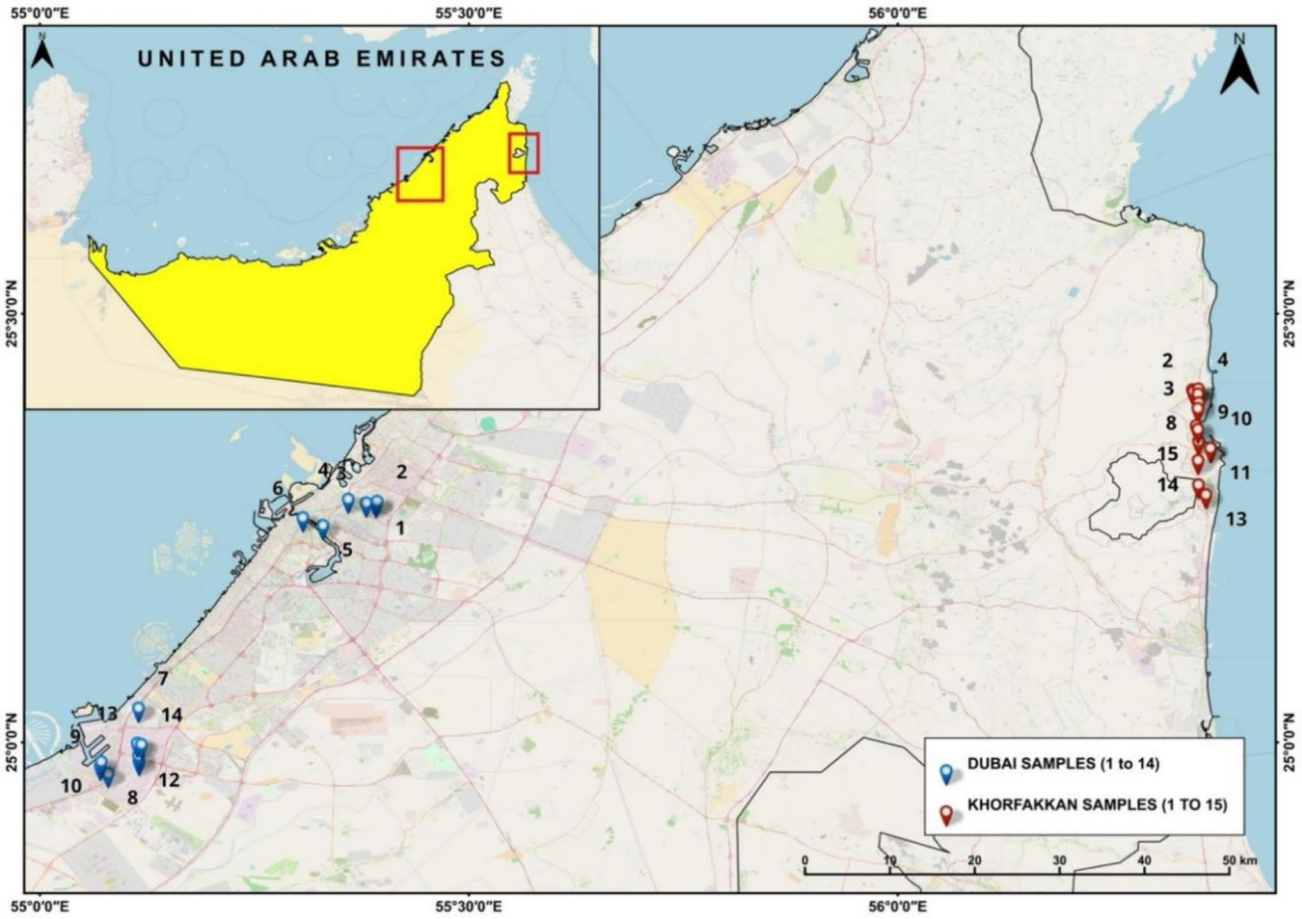
Location map of roadside dust samples collected from urban area- Dubai and mountainous area- Khor Fakkan, UAE

Road dust samples were systematically collected from twenty-nine locations in Dubai and Khor Fakkan City (Fig. 1), covering low- and high-density traffic roads, industrial zones, hospitals, parks, and parking areas, to capture exposure across a diverse population, including adults, patients, and children. The sampled locations—such as Albidaya Industrial, Hay Alzubara, and Rugaylat Road in Khor Fakkan, and Al Qouz Industrial, Al Qusais Industrial, Jebel Ali Industrial, and Deira in Dubai—have typical vehicle speed limits ranging from 25 to 60 km/h. On major roads like the Sharjah–Khor Fakkan and Dubai–Sharjah highways, speeds reach 100–120 km/h, with reductions near urban or mountainous areas. Wind speeds in these regions also vary seasonally, ranging from 8 to over 18 km/h in Khor Fakkan and 7 to over 15 km/h in Dubai.

Each sample (~ 200–300 g) was stored in an airtight container with appropriate labeling. Subsequently, the samples were dried at 105 °C and cooled in a desiccator. Before sieving, the samples were meticulously cleaned of extraneous materials such as stones, leaves, plastics, and other coarse debris. After passing through a 63 μm sieve, 0.2 g of each sample was accurately weighed and transferred into 100 ml Teflon digestion vessels. A 1:1 mixture of HNO₃ and HCl was added to the vessels before undergoing microwave digestion (USEPA 3050B, 1996). The samples were pre-heated at approximately 95 °C for 5 min. After cooling, 3 ml of 10% H_2_O_2_ was added for oxidation, and the vessels were allowed to stand until all fumes had dissipated. The Teflon digestion vessels were then hand-tightened and placed in the microwave digestion system for a 30-min program. After digestion, the vessels were cooled, and each sample was diluted with deionized ultra-pure water (Milli-Q Integral 5, resistivity 18 MΩ·cm). The samples were then filtered using a 0.45 μm PVDF syringe filter. Heavy metal analysis (Cd, Cr, Cu, Ni, Pb, Co, Ba, Fe, Mn, and Zn) was performed using an ICP-OES instrument (PerkinElmer Avio 200, USA), equipped with a wavelength range of 120 nm to 850 nm and an autosampler. The instrument was calibrated using certified multi-element standards (Merck Multi-element Standard IV, CAS No. 111355), covering a concentration range of 0.10 ppm to 10 ppm. Calibration curves were constructed for each element of interest. The digested and filtered samples were analyzed using an Optical Emission Spectrophotometer (OES) under the following instrumental parameters: The ICP-OES was operated at a power of 1 kW, with both axial (A) and radial (R) view modes employed for optimal detection of elements. The plasma gas flow rate was maintained at 15 L/min, with an auxiliary flow rate of 0.5 L/min and a nebulizer flow rate of 0.5 L/min. The sample uptake rate was set to 1 ml/min, with a rinse time of 22 s between samples. The stabilization time before measurement was 12 s. Additionally, the pump operated in fast mode with a flow rate of 4 ml/min to ensure efficient sample introduction. The performance of the ICP-OES method was evaluated through system suitability parameters, which are summarized in Table 1. These include the correlation coefficient (r), R-squared (r^2^), calibration curve slope and intercept, residual standard deviation (σ), and calculated method detection (LOD) and quantitation (LOQ) limits for each target metal. All calibration curves showed excellent linearity (r ≥ 0.9984), indicating high accuracy and sensitivity of the method across the concentration range.

**Table 1:** System suitability parameters for ICP-OES calibration, including linearity (r, r^2^), slope, intercept, residual standard deviation (σ), method detection limits (LOD), and method quantitation limits (LOQ) for each metal

Metal	Correlation coefficient, *r*:	R-squared, *r*2:	Slope, m:	Y-intercept, c:	Residual Stdev (σ)	Method limit of detection (LOD) ppm	Method limit of quantitation (LOQ) ppm
Cd	0.9996	0.9992	362,227.4091	− 46,503.97453	18,702.7445	0.17 ppm	0.52 ppm
Cr	1.0000	0.9999	382,928.3705	− 17,218.8687	2328.0707	0.02 ppm	0.06 ppm
Cu	1.0000	0.9999	577,829.5514	− 52,720.90537	14,454.3337	0.08 ppm	0.25 ppm
Ni	0.9996	0.9991	135,074.0807	− 21,661.10143	3491.9729	0.09 ppm	0.25 ppm
Pb	0.9984	0.9968	21,007.8663	1454.40346	795.4512	0.12 ppm	0.38 ppm
Co	0.9999	0.9999	165,407.5678	− 9108.31321	3994.2718	0.08 ppm	0.24 ppm
Ba	0.9998	0.9996	5,314,839.7769	66,260.71028	65,152.7390	0.06 ppm	0.19 ppm
Fe	0.9995	0.9991	387,591.2833	11,721.1371	20,644.1496	0.18 ppm	0.53 ppm
Mn	0.9999	0.9998	2,277,558.3558	28,399.90058	142,834.5578	0.21 ppm	0.63 ppm
Zn	0.9997	0.9994	392,285.7223	− 26,356.88251	11,608.1398	0.10 ppm	0.30 ppm

To ensure data reliability, quality control measures were implemented. These included the analysis of reagent blanks to check for contamination, the preparation and analysis of replicate samples to assess precision, and the analysis of certified reference materials (CRM—LGC batch no: 1288797) to verify method accuracy. Additionally, a 5 ppm spiked standard run was performed after every 10 analyses to monitor method accuracy. All recovery values were within the acceptance criteria as per ICH method validation guidelines (Q2(R1)), confirming that all results were accurately measured by the instrument.

Quality Control: To ensure data reliability, quality control measures were implemented. These included the analysis of reagent blanks to check for contamination, the preparation and analysis of replicate samples to assess precision, and the use of certified reference materials (CRM – LGC, batch no: 1288797) to verify method accuracy. Additionally, a 5 ppm spiked standard was run after every 10 analyses to assess the accuracy of the method. All recovery values obtained were within the acceptable criteria outlined in the ICH method validation guideline (Q2(R1)), confirming that the results were accurately measured by the instrument (Table 2).

**Table 2:** Percentage recovery of quality control elements in spiked samples

Element	Cd	Cr	Cu	Ni	Pb	Co	Ba	Fe	Mn	Zn
% Recovery	97.98%	95.65%	96.44%	98.65%	95.65%	95.99%	97.55%	102.76%	96.76%	101.55%

### Data Analysis Methodology

To facilitate sample identification, samples collected from Dubai were labeled D1 through D14, while those collected from Khor Fakkan were labeled KF1 through KF15.

### Pollution Indices

To estimate the pollution levels or enrichment with various metals based on the elements’ concentration in the dust, two types of indexes were calculated:

1. Individual: Geo-accumulation index (*I*_geo_), the pollution index (PI), the enrichment factor (EF), and the contamination factor (CF).
2. Complex: Obtained by aggregating the individual ones. They are the Pollution Load Index (PLI), Nemerow Index (${\text{PI}}_{\text{Nem}}$), Combined Pollution Index (CPI), Contamination Degree (CD), and Modified degree of contamination (*m*CD).

Geo-accumulation index (*I*_geo_) of the element *i* is defined by the equation (Muller 1969; Kowalska et al. 2018):

1 $$I_{{{\text{geo}}}} = \log_{2} \left( {C_{i} /\left( {1.5CB_{i} } \right)} \right),$$

where ${C}_{i}$ and ${CB}_{i}$ are the element’s concentrations in the sample and background, respectively. The background concentrations utilized in this study were taken from (Lindsay 1979).

Seven pollution classes are considered based on some boundary values: extreme if ${I}_{\text{geo}}\ge$ 5, strong to extreme if ${I}_{\text{geo}} \in \left[4\right.,5),$ strong ${I}_{\text{geo}} \in \left[3\right.,4),$ moderate to strong when ${I}_{\text{geo}} \in \left[2\right.,3)$, moderate when ${I}_{\text{geo}}\in \left[1\right.,2)$, unpolluted to moderately polluted if ${I}_{geo}\in \left[0\right.,1)$, unpolluted if ${I}_{\text{geo}}<0$.

The Pollution index (PI) of the element *i*, ${\text{PI}}_{i},$ is defined by (Kowalska et al. 2018):

2 $${\text{PI}}_{i} = C_{i} /{\text{CB}}_{i} .$$

Values of the index less than 1 indicate no pollution. Values in the intervals [1, 2), [2,3), [3,5) show low, moderate and strong pollution, respectively, whereas values higher than or equal to 5, point out on very accentuated pollution of the soil.

The Enrichment factor (*EF*) with the element *i*,${EF}_{i},$ is given by (Sutherland 2000):

3 $${\text{EF}}_{i} = {\text{PI}}_{i} /{\text{PI}}b_{i}$$

where ${PI}_{i}$ have the meaning in (2) and ${PIb}_{i}$ is the corresponding pollution index of the background element.

*EF* < 2 indicates a deficient to minimal enrichment, values in the interval [2,5) –moderate, [5, 20)—significant, between 20 and 40, very high, and at least 40 extremely high enrichment in a particular element.

Contamination factor (CF) for a metal *j* $({\text{CF}}_{j})$ is determined as the ratio between the mean concentration of *j* in at least five samples $({C}_{avj})$ and the reference concentration of *j* from the pre-industrial era $({C}_{pj})$ (Håkanson 1980), i.e.,

4 $${\text{CF}}_{j} = C_{{{\text{avj}}}} /C_{{{\text{pj}}}}$$

The reference values (µg/g) are 1—Cd, 90—Cr, 50—Cu, 68—Ni, 70—Pb, 175—Zn, and 850—Mn (Håkanson 1980). The intervals [0,1), [1,3), [3,6) associated with ${CF}_{j}$ indicate low, moderate, and high contamination.

Values greater than 6 show a very high contamination.

Complex indexes, built from the simple ones, are utilized to estimate pollution with many elements at a site.

Pollution Load Index (PLI) was calculated by the formula (Tomlinson et al. 1980):

5 $${\text{PLI}} = \left( {\mathop \prod \limits_{j = 1}^{n} {\text{PI}}_{j} } \right)^{1/n} ,$$

where *n* is the number of metals. PLI < 1 signifies mean perfection, whereas equal to 1 or greater than 1 show baseline contamination or contamination respectively.

$\text{Nemerow Index }({PI}_{Nem})$ is defined by (Gong 2008):

6 $${\text{PI}}_{{{\text{Nem}}}} = \sqrt {\left[ {\overline{{{\text{PI}}}}^{2} + {\text{PI}}_{\max }^{2} } \right]/2}$$

where ${PI}_{max}$ is the maximum *PI* of the analyzed metals, and $\overline{PI }$ is their average.

The reference intervals for ${PI}_{Nem}$ are [0,0.7), [0.7,1), [1,2), [2,3), $\ge 3$, indicating the pollution absence, a warning level, slight pollution, moderate pollution, and heavy pollution, respectively.

Combined Pollution Index (CPI) is computed by the formula (Nazzal et al. 2023; Inengite et al. 2015):

7 $${\text{CPI}} = \frac{1}{n}\mathop \sum \limits_{j = 1}^{n} {\text{PI}}_{j} .$$

The reference values are kept those for ${\text{PI}}_{\text{Nem}}$. $\text{CPI}>1$ indicates a high contamination (Inengite et al. 2015).

Contamination Degree (CD) is obtained by summing the values of the contamination factors of all the studied metals. The Modified degree of contamination (*m*CD) is defined as the average of the *CF*s. The reference intervals for different contamination classes are those for the CF multiplied by the number of metals (Gong 2008).

### Assessing the Pollution Extent at the Local Level

The assessment of the pollution at the local level was done based on the method proposed by Bărbulescu (2016). Modeling was performed using the series of EF indices determined at each location. Similar approach can be used for the serries of PI or *I*_geo_ computed for Dubai and Khor Fukkan series. The steps of the algorithm are presented in the following.

Form the matrix of the *EF* indices – each column contains the indices computed at each sampling site (for example, column 2 contains the indices computed for D2). The matrix can be generically denoted by *M*_EF_.

For each row of *M*_EF_, perform the following operations:

- Compute the minimum, maximum, and the difference between them.
- Divide the intervals between minimum and maximum into a number (*m*) of intervals of the same length, so that none of the resulted intervals is void. In this application, after the analysis of the data series, *m* was chosen equal to 3.
- Assign to each sub-interval its frequency (i.e., the number of the values of the index situated in that sub-interval).
- Define the representative sub-interval, which is the one with the highest frequency. If there are many sub-intervals with the same maximum frequency, select that one whose mean, computed as the average of the vales inside it, is the closest to the mean of the values on the same row*.*

### Clustering the Data Series

Data series in each zone were grouped in the clusters using the k-medoid algorithm (Kaufmann and Rousseeuw 1986). It represents each cluster by one of its actual data points, called "medoid." The medoid is the point within the cluster whose average dissimilarity to all other points in the same cluster is the smallest. K-medoids seek to minimize the sum of dissimilarities between each point and its corresponding medoid, i.e.,:

8 $$\arg \min_{S} \mathop \sum \limits_{i = 1}^{k} \mathop \sum \limits_{{x \in S_{i} }} d\left( {x,m_{i} } \right)$$

where ${m}_{i}$ is the medoid of cluster ${{\varvec{S}}}_{i}$ and $d(x,{m}_{i})$ is the dissimilarity between point $x$ and the medoid.

The algorithm has the following steps:

- Define the number of clusters *k*.
- Randomly select *k* initial centroids from the data.
- Assign each data point ${x}_{i}$ to the nearest medoid to achieve minimum in (8)
- Update the centroid so that the new centroid of each cluster is the average value of the data points assigned to that cluster.
- Repeat the assignment and medoid update until convergence or the stop criteria is reached.

This method’s gives an advantage of providing consistent results when working with non-spherical clusters compared to k-means and yield stable outcomes.

The majority selection criterion was used to choose the number of clusters, *k*, after running the set of algorithms implemented in the NbClust package in R (Charrad et al. 2022).

The level of connectivity inside the clusters was analyzed using the Connectivity, Dunn, and Silhouette indexed (Brock et al. 2008), and the clusters stability by the Jaccard index (Hennig 2019, 2020).

### Principal Components Analysis

The principal component analysis (PCA) is used to diminish the number of variables. It replaces the initial variables with Principal Components (PC) built as their linear combinations. The extracted PCs should explain more than four fifth of the initial variables’ variance (Jolliffe 2014). The PC selection can be made using various methods, including the Catell Scree Plot, explained Variance, and Kaiser criterion (Jolliffe 2014; Cattell 1966; Kaiser 1960). The PCA was analyzed using the R 4.3.1 software (https://cran.r-project.org/).

## Results and Discussion

### Spatial Distribution of Metals in Street Dust

The comparative analysis of metal concentrations in Dubai and Khor Fakkan samples reveals significant variations across different locations, indicating potential localized sources of pollution in both regions. The basic statistics of metal concentrations in road dust samples from Khor Fakkan and Dubai are presented in Tables 3 and 4, respectively. In Dubai, Cd levels are notably high in D1 and D12, both around 44 mg/kg, suggesting specific areas of contamination, while D7 shows the lowest concentration at 13.96 mg/kg. Similarly, in Khor Fakkan, Cd levels are highest in KF2 (47.99 mg/kg) and lowest in KF3 (18.05 mg/kg). Cr concentrations vary widely in both regions, with D14 having the highest at 207.30 mg/kg and D1 the lowest at 38.19 mg/kg, while KF6 has the highest Cr concentration at 153.49 mg/kg and KF3 the lowest at 52.80 mg/kg. Cu levels are highest in D6 at 154.03 mg/kg and lowest in D12 at 26.97 mg/kg, whereas in Khor Fakkan, Cu levels peak in KF12 at 102.77 mg/kg and are lowest in KF2 at 35.95 mg/kg. Ni shows significant hot spots in both regions, with D8 at 543.20 mg/kg and KF2 at 2621.05 mg/kg.

**Table 3:** Basic statistics of the metal contents of road dust obtained from the Khor Fakkan (KF) Samples (mg/kg)

KF	Cd	Cr	Cu	Ni	Pb	Co	Ba	Fe	Mn	Zn
Mean	33.219	104.14	59.970	1325.4	103.92	33.192	57.679	17,051	233.66	712.86
Min	18.047	52.802	35.952	145.44	17.541	14.608	35.971	6715.3	158.56	435.64
Max	47.993	153.48	102.76	2621	315.06	70.842	99.714	20,982	331.01	1802.2
Std dev	10.266	25.774	21.836	502.13	78.138	16.504	16.907	3465.1	42.647	346.69
Skew	0.088	− 0.109	1.011	0.425	1.394	1.515	1.159	− 1.973	0.522	2.465
Kurtosis	− 1.595	0.057	− 0.558	4.58	2.996	1.145	1.476	5.401	0.995	7.218

**Table 4:** Basic statistics of the metal contents of road dust obtained from the Dubai Samples (mg/kg)

Dubai	Cd	Cr	Cu	Ni	Pb	Co	Ba	Fe	Mn	Zn
Mean	25.885	89.152	62.68	201.34	33.748	28.767	94.57	10,915	197.31	313.39
Min	13.959	38.187	26.971	105.68	14.541	15.448	44.27	4264.1	139.31	120.75
Max	44.035	207.29	154.03	543.20	72.194	66.35	168.4	17,976	273.23	587.87
Std dev	11.8372	52.7611	39.0842	116.153	15.410	17.536	33.03	4812.5	39.385	166.82
Skew	0.718	1.54	1.606	2.208	1.086	1.57	0.775	0.191	0.497	0.242
Kurtosis	− 1.369	1.534	1.708	5.588	1.651	1.244	1.077	− 1.582	− 0.796	− 1.557

Pb concentrations are highest in D9 at 72.19 mg/kg and lowest in D12 at 14.54 mg/kg, while in Khor Fakkan, Pb levels are highest in KF2 at 315.06 mg/kg and lowest in KF5 at 20.26 mg/kg. Co levels are highest in D1 at 66.35 mg/kg and lowest in D12 at 15.45 mg/kg, with Khor Fakkan showing the highest Co levels in KF2 at 70.84 mg/kg and the lowest in KF3 at 14.61 mg/kg. Ba concentrations are highest in D9 at 168.35 mg/kg and lowest in D2 at 44.27 mg/kg, while in Khor Fakkan, Ba levels peak in KF13 at 99.71 mg/kg and are lowest in KF2 at 35.97 mg/kg. Fe levels show a wide range in both regions, with D6 having the highest at 17,976.65 mg/kg and D3 the lowest at 4264.01 mg/kg, and Khor Fakkan’s highest Fe levels in KF1 and KF4 at 20,982.90 mg/kg and the lowest in KF3 at 6715.35 mg/kg. Mn concentrations are highest in D5 at 273.23 mg/kg and lowest in D8 at 139.31 mg/kg, while in Khor Fakkan, Mn levels are highest in KF2 at 331.01 mg/kg and lowest in KF8 at 176.57 mg/kg. Zn levels are highest in D5 at 587.88 mg/kg and lowest in D14 at 120.76 mg/kg, with Khor Fakkan showing the highest Zn levels in KF1 at 1802.02 mg/kg and the lowest in KF11 at 435.64 mg/kg.

**Fig. 2:**
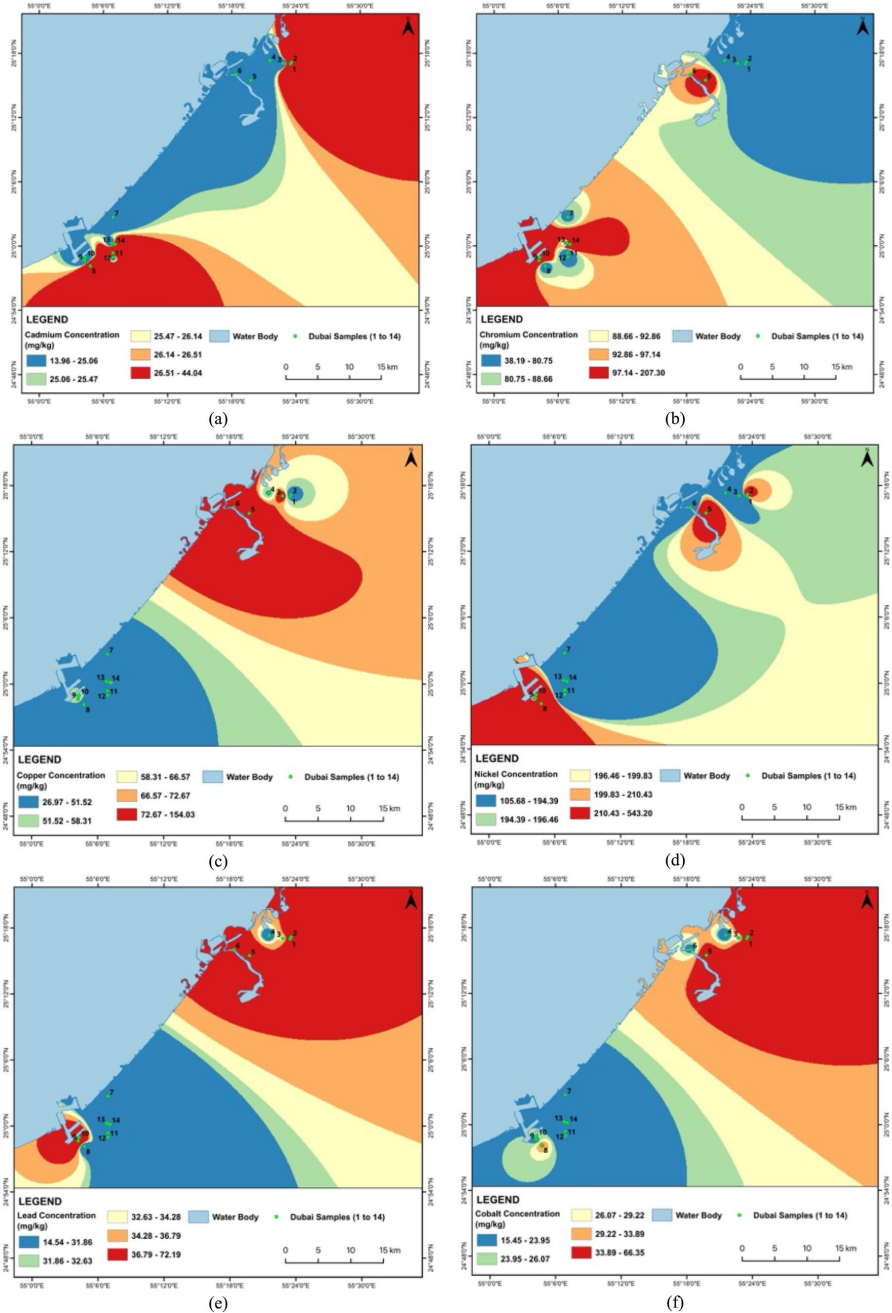

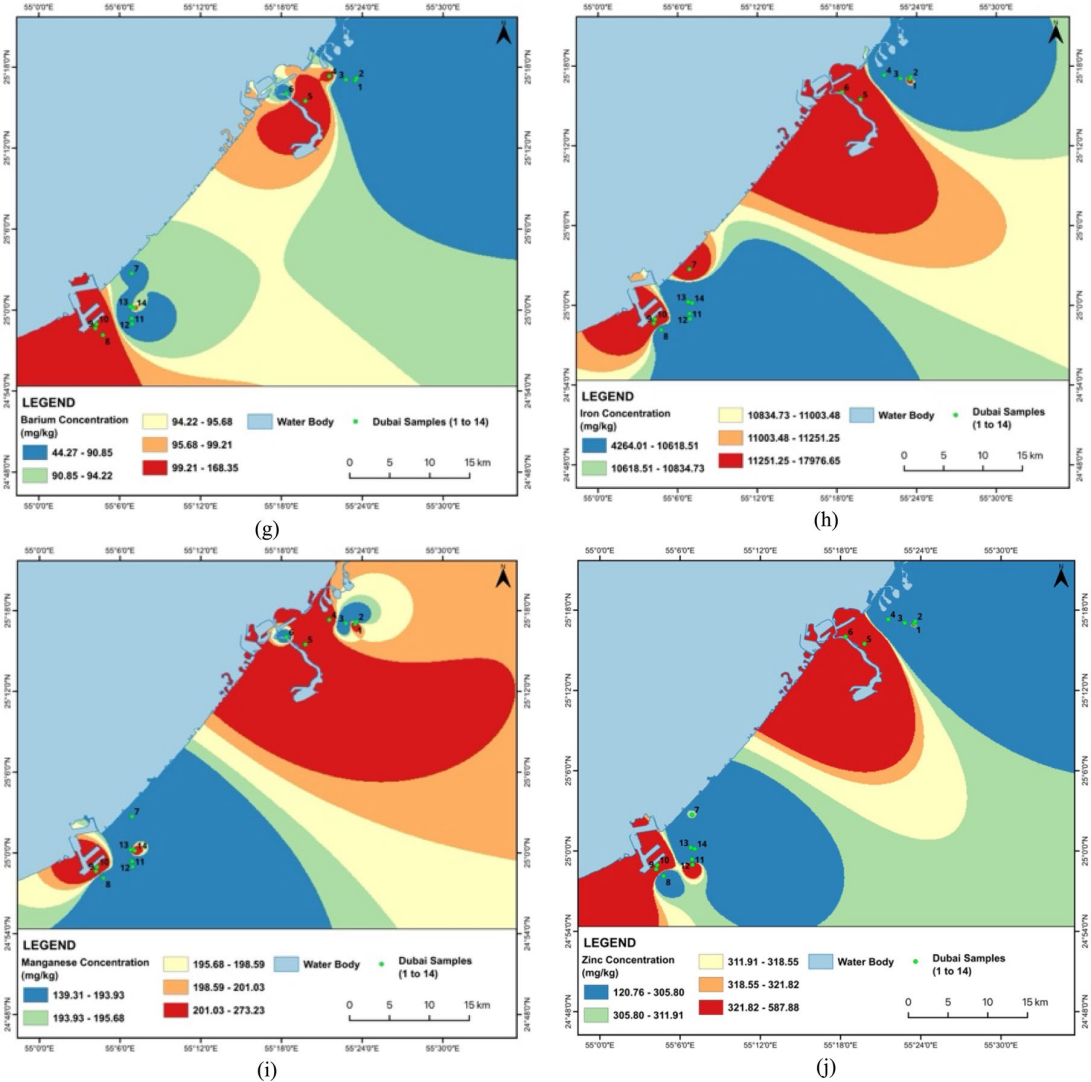
Spatial distribution for heavy metals concentration for **a** Cd, **b** Cr, **c** Cu, **d** Ni, **e** Pb, **f** Co, **g** Ba, **h** Fe, **i** Mn and **j** Zn in street dust sample from Dubai, UAE

**Fig. 3:**
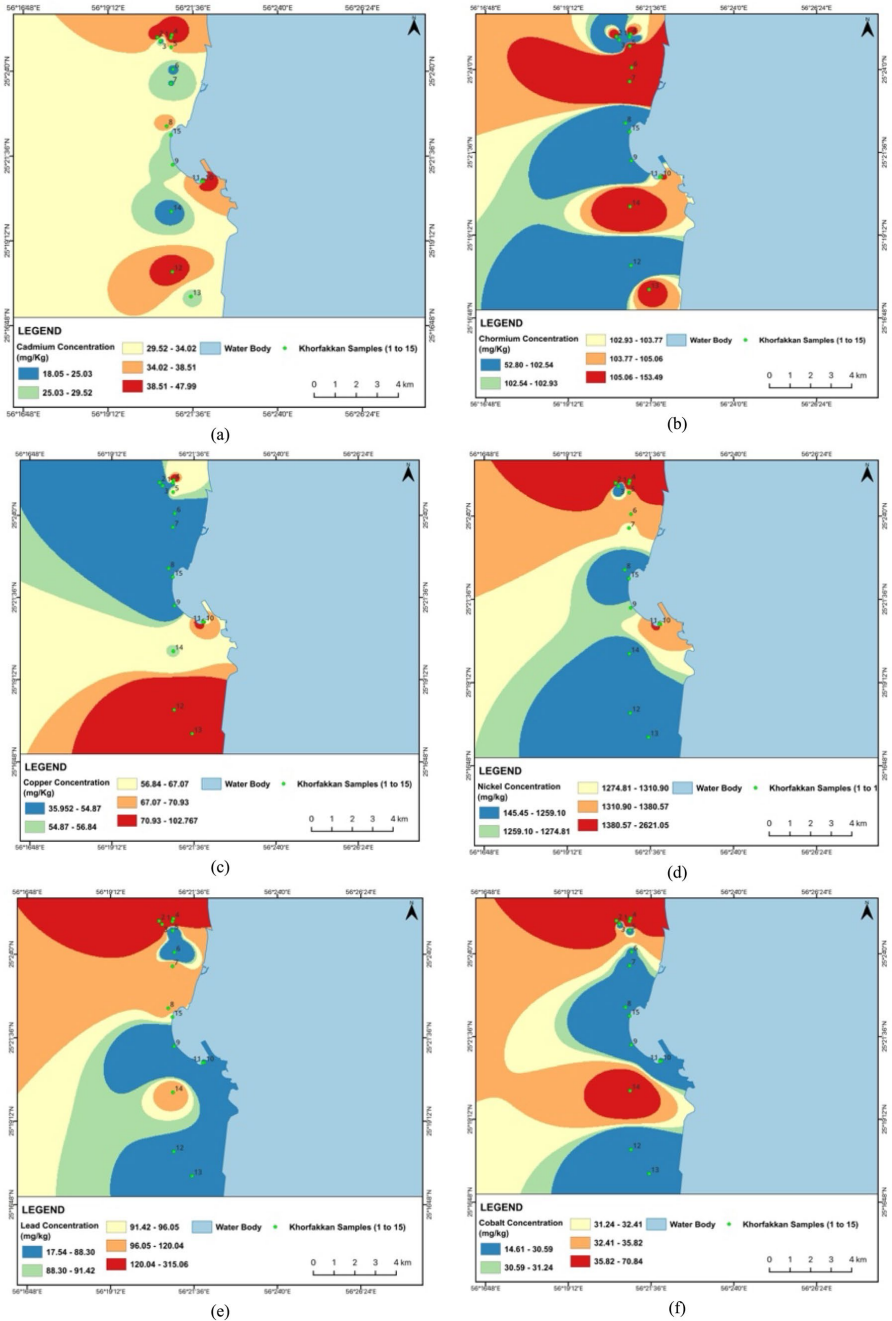

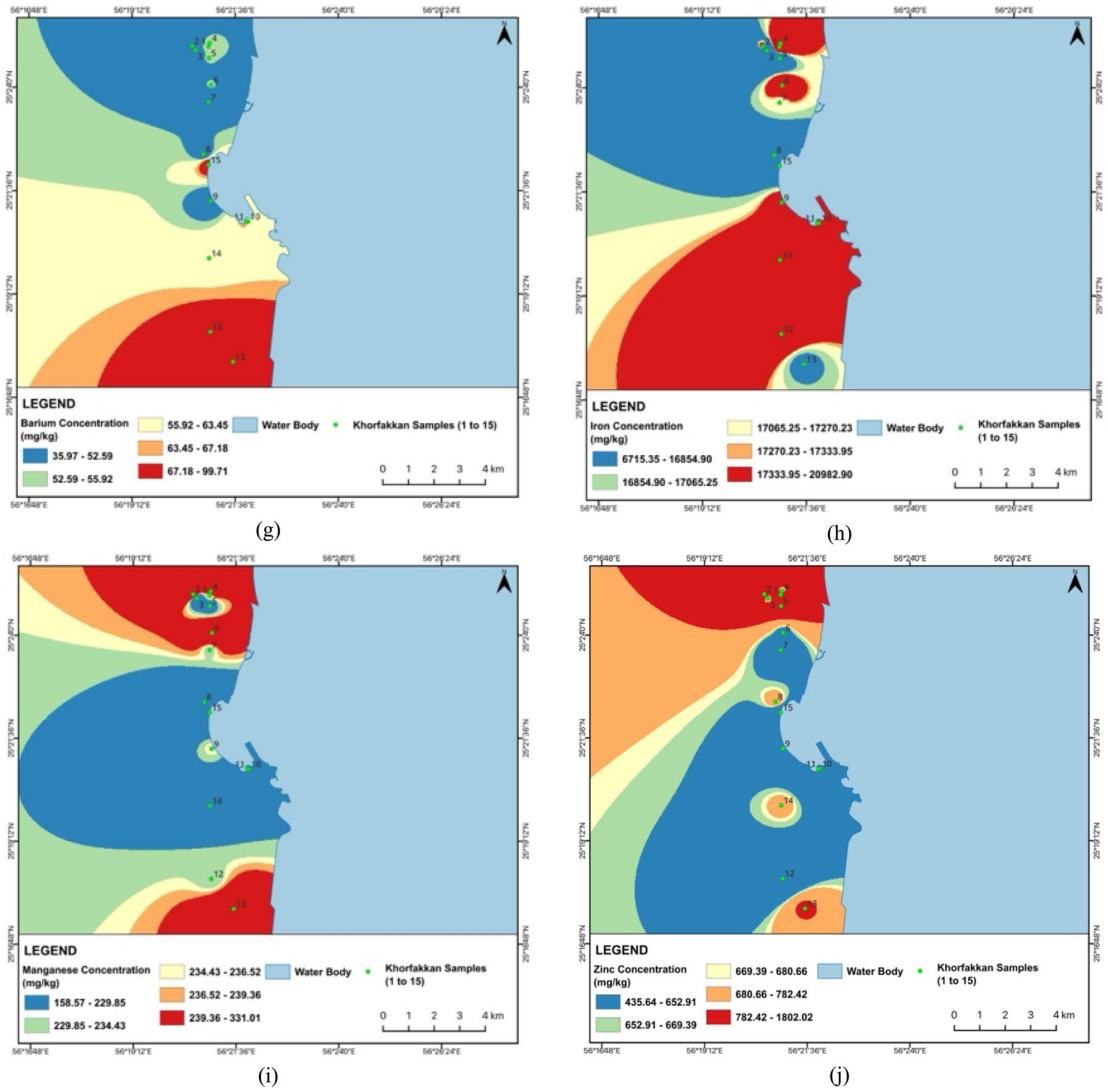
Spatial distribution for heavy metals concentrations: **a** Cd, **b** Cr, **c** Cu, **d** Ni, **e** Pb, **f** Co, **g** Ba, **h** Fe, **i** Mn and **j** Zn in street dust sample from Khor Fakkan, UAE

The Cd levels are alarmingly high across all samples, far exceeding the standard value of 1.1 mg/kg, with Khor Fakkan samples showing slightly higher concentrations (18.05–47.99 mg/kg) than Dubai samples (13.96–44.03 mg/kg). Cr levels are generally within acceptable limits, except for D14, which approaches the threshold, and Khor Fakkan samples showing higher concentrations (52.80—153.49 mg/kg) compared to Dubai (38.19–207.30 mg/kg). Cu and Ni concentrations are notably high in several samples from both regions, particularly D6 and D8, and KF12 and KF2, respectively, suggesting industrial discharge or other anthropogenic sources. Pb levels are relatively high in D9 and KF2, while Co concentrations exceed the standard in multiple samples from both regions. Ba, Fe, and Mn levels are within safe limits, indicating no immediate concern. Zn concentrations are elevated in several samples, with D5 and KF1 showing the highest levels. Figures 2 and 3 show the spatial distribution of heavy metal concentrations in street dust samples collected from Dubai and Khor Fakkan, UAE. The maps were prepared using ArcGIS software to facilitate visualization and enhance understanding of heavy metal distribution across the region.

The analysis reveals that certain metal concentrations in Khor Fakkan are extremely higher compared to Dubai, indicating significant localized pollution. Notably, Ni levels in Khor Fakkan, particularly in KF2 (2621.05 mg/kg), are substantially higher than in Dubai, where the highest concentration is 543.20 mg/kg. This suggests a major hot spot for nickel contamination in Khor Fakkan, likely due to intensive industrial activities or vehicular emissions. Similarly, Pb concentrations in Khor Fakkan, especially in KF2 (315.06 mg/kg), are much higher than in Dubai (72.19 mg/kg), which could be attributed to higher traffic emissions or more extensive industrial activities. Zinc (Zn) levels are also significantly elevated in Khor Fakkan, with KF1 reaching 1802.02 mg/kg, compared to the highest value in Dubai (587.88 mg/kg).

### Analysis of the Pollution Indexes

**Fig. 4:**
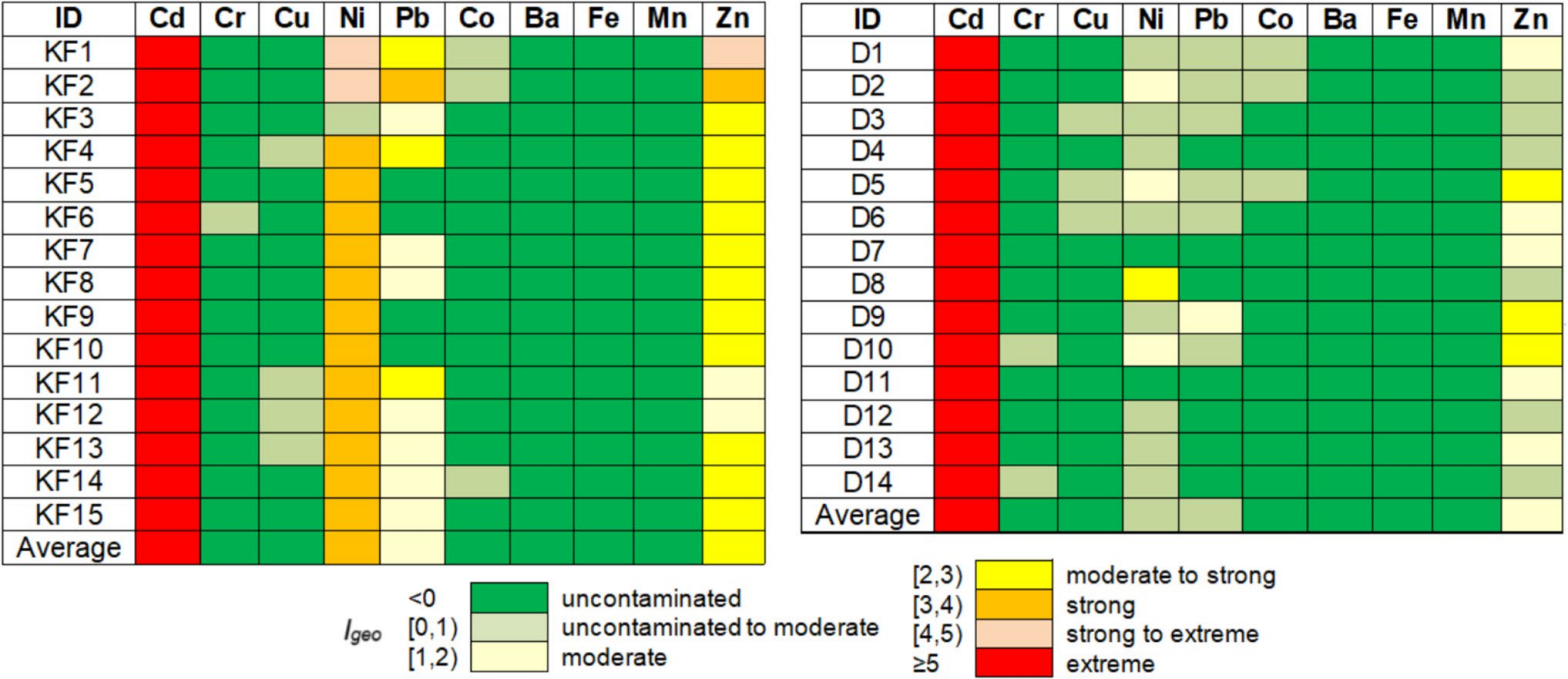
Degree of pollution based on the values of ${I}_{geo}$

**Fig. 5:**
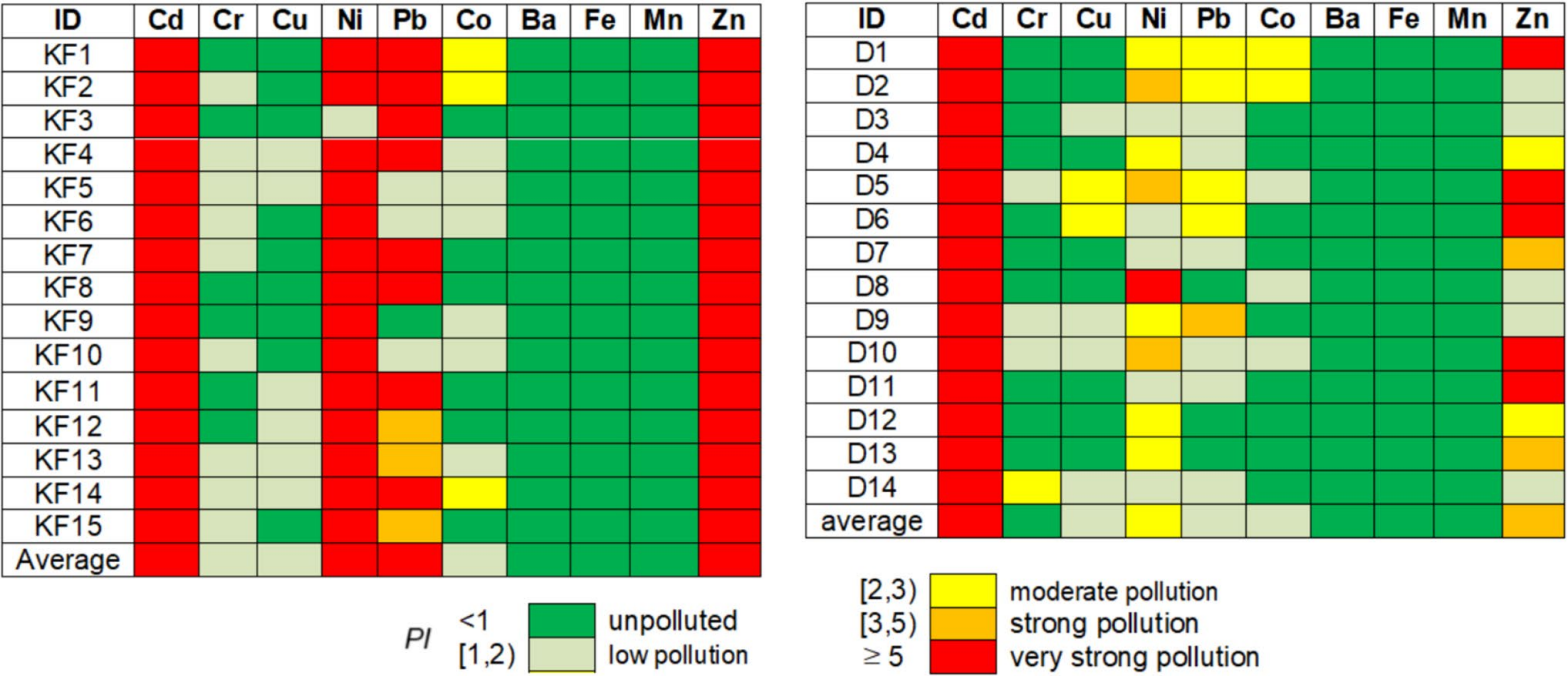
Degree of pollution based on the *PI* values

**Fig. 6:**
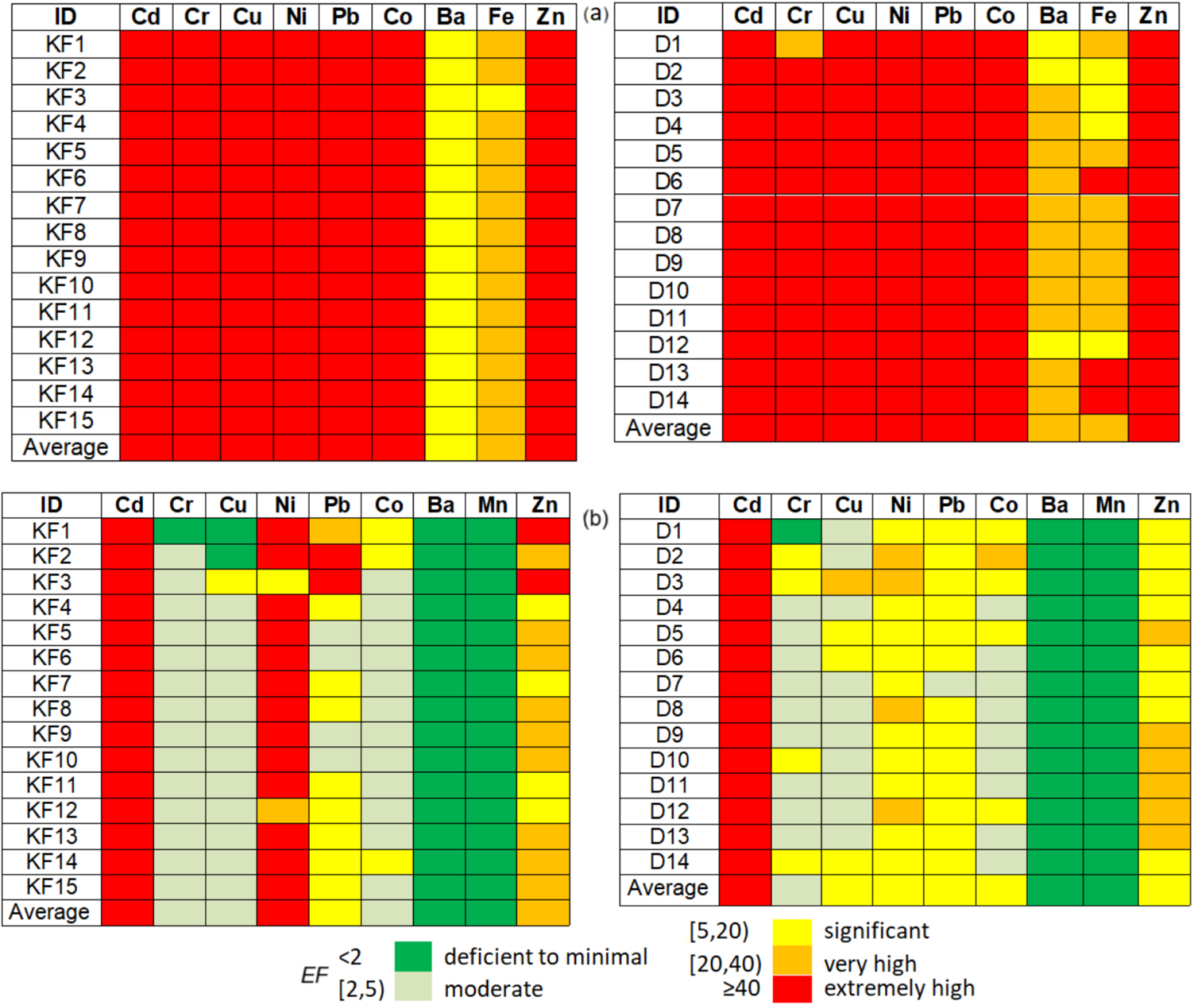
Enrichment factor (*EF)* with elements with respect to **a** Mn and **b** Fe

Figures 4, 5 and 6 are the graphical representations of the pollution levels based on the ${I}_{\text{geo}}, \text{PI}$ and EF.

According to ${I}_{\text{geo}}$ values (Fig. 4), there is no contamination with Ba, Fe, and Mn. The soils are uncontaminated or uncontaminated to moderately contaminate with Cu, Cr, and Co at all Khor Fakkan and Dubai sites. The highest contamination is with Ni (strong or strong to extreme) and Zn (in majority moderate to strong) in Khor Fakkan. In Dubai, the highest contamination is with Zn in D5, D9, D10, and Ni in D8. With respect to the *PI* (Fig. 5), there is no pollution with Ba, Fe, and Mn. The soils are unpolluted or low polluted with Cr, Cu, and Co at all sites in Khor Fakkan and Dubai, excepting KF1, KF2, KF 14, D1, and D2 (moderately polluted with Co), D5 and D6 (moderately polluted with Cu). Very strong pollution with Cd, Zn and Ni is recorded in Khor Fakkan, whereas in Dubai, it is found with Cd, with Zn at D1, D5, D6, D11, and D12, and Ni in D8.

**Fig. 7:**
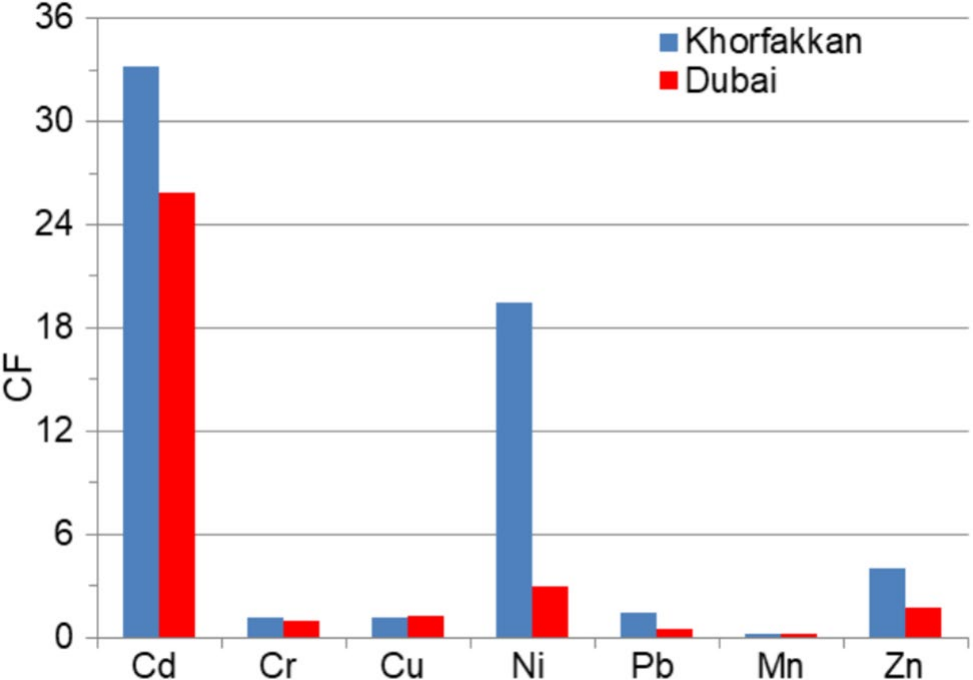
*CF* of various metals

The *EF* computed with respect to Mn (Fig. 6(a)) indicates a significant enrichment with Ba at KF1-KF15, D1, D2, D12, and with Fe at KF3, D2-D4, and D12. Very high enrichment is noticed with Fe at all locations in Khor Fakkan but KF3, D1, D5, D7-D11, Cr at D1, and Ba at D3-D11, D13, and D14. Extremely high enrichment with all the others are observed. *EF* computed with respect to Fe (Fig. 6(b)) gives various results: extremely high enrichment appears only with Cd (all sites) and Ni (in Khor Fakkan) whereas deficit to minimal is recorded with Ba and Mn (at all sites). The enrichment of Cr, Cu, and Co is moderate at most sites, while Pb (and Ni) shows significant to very high enrichment in Dubai. The variation in contamination factors is illustrated in Fig. 7. Contamination levels are low for Cr, Pb, and Mn in Dubai, as well as Mn in Khor Fakkan; moderate for Cr, Cu, and Pb in Khor Fakkan and Cu, Zn, and Ni in Dubai; high for Zn in Khor Fakkan; and very high for Cd in both regions and Ni in Dubai.

The PLI values varied in the intervals 0.868–2.165 at Khor Fakkan, and 0.642 -1.499 at Dubai, indicating perfection at KF3, D2-D4, and D11-D14, and contamination in the other sites. Since the PI values for Cd are very high compared to the others, removing them and recomputing, we obtained indices greater than 1 only for KF1, KF2, KF4, KF13, KF14. The ${PI}_{Nem}{\prime}$ values were greater than 73.08 at Khor Fakkan and 49.68 at Dubai. We consider them irrelevant due to the very high values of *PI* for Cd. Removing the *PI* for Cd and recomputing, ${PI}_{Nem}$ were between 6.21 and 25.32 at Khor Fakkan, and in the interval (1.63, 5.77) at Dubai. Thus, KF1-KF15, D1, D5, D6, D8-D11 are heavily polluted, whereas D2, D7, and D13 are moderately polluted, and D3, D4, D13, and D14 are slightly polluted.

*CPI* computed with or without considering the *PI* for Cd indicates a high contamination in Khor Fakkan. *CD*, obtained by summing up all *CF*, had values of 60.90 at Khor Fakkan and 33.60 at Dubai, indicating a very high degree of contamination in the first case and a high level in the second. *mCD* is 8.70 and 4.80, respectively, showing that the soil in Khor Fakkan is very highly contaminated and that in Dubai is highly contaminated.

**Fig. 8:**
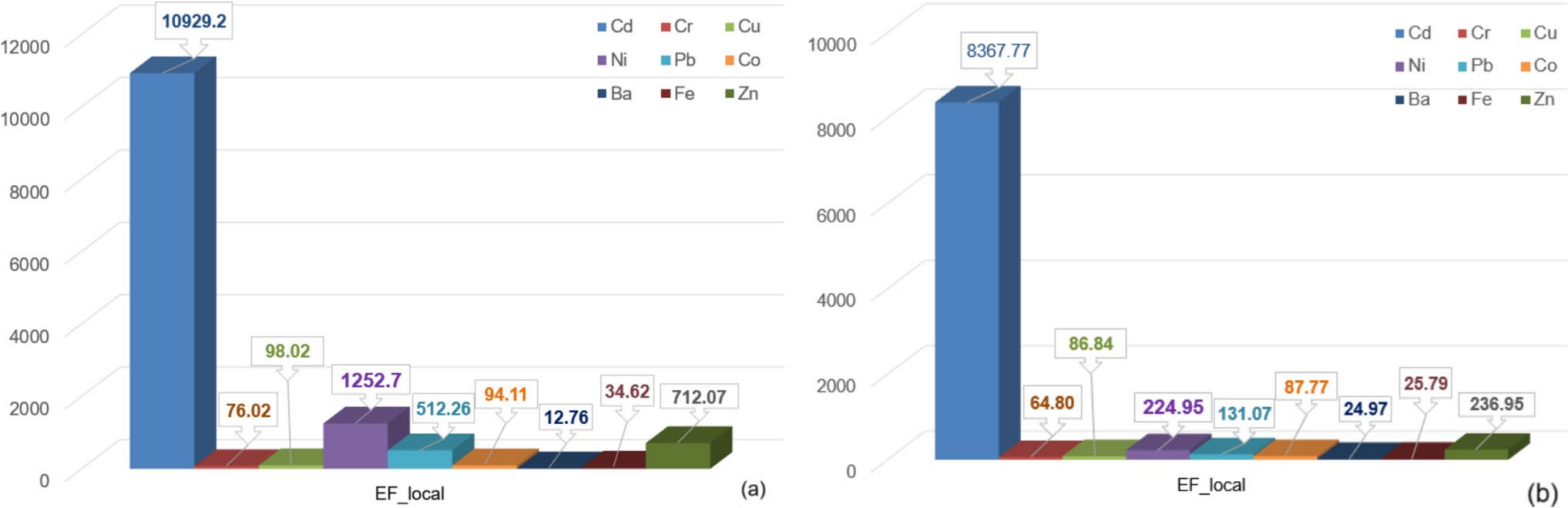
*EF_local* for **a** Dubai zone and **b** Khor Fukkan zone

Applying the method from the Sect. 2.3.2 to the series of *EF* indexes, separate for Dubai and Khor Hukkan, we determined the representative local level of EF shown in Fig. 8. Since the sampling sites are close to each other in each area, the above representation has the advantage to emphasize the representative level of pollution indicators without analyzing a high number of values and having a synthetic image. It is shown that the highest enrichment is that with Cd, followed by Ni, in both regions. Moreover, the enrichment at the local level is significantly lower in Khor Fukkan compared to Dubai. Similar analyses ca be performed for the other sets of indexes, leading to the same conclusion.

To complete these statistics, we might consider the average values of the concentration series of each element at KF and Dubai, respectively, then we used a non-parametric ANOVA to determine if there is a difference between these series. Whereas correct from a mathematical viewpoint, from a physical viewpoint it is not correct because we obtain series containing concentrations of different elements. Therefore, to avoid this inconvenience, the ANOVA test was performed not on the series itself but on the indicator’s series, i.e., Igeo, PI, EFMn, and EFFe. The p values computed in these tests were all less than 0.05 (the significance level) indicating a significant difference between the pollution levels at KF and Dubai.

Similar tests have been done to compare the contamination levels with various elements. The p values in the nonparametric ANOVA tests are presented in the Table 5. Since the p values are smaller than 0.05, the hypothesis that the series of indices are alike can be rejected.

**Table 5:** Pollution indices (Igeo, PI, EF for Mn and Fe) in road dust from Khor Fakkan (KF) and Dubai, with significantly different values (p < 0.05) based on nonparametric ANOVA tests

Pollution index	KF	Dubai
Igeo	4.55E-26	3.65E-24
PI	3.26E-24	1.97E-23
EF_Mn_	1.31E-25	1.21E-22
EF_Fe_	1.44E-25	2.18E-22

### Clustering the Data Series and Principal Component Analysis (PCA)

After applying the selection procedures implemented in the NbClust package, the best number of clusters for Khor Fakkan was determined to be 3 (selected by 11 algorithms), followed by 2 (selected by 9 algorithms). For Dubai, the best number of clusters were 3 and 6 (each selected by 6 algorithms) and 2 (selected by 5 algorithms).

The analysis of the connectivity inside the clusters and the clusters’ stability performed after running the k-medoids algorithm with *k* = 3, 6, and 2 for Khor Fakkan indicates that the best performances are obtained in the first and third cases. The highest connectivity inside the clusters was found for *k* = 2. The groups found are stable when *k* = 6 (the AJ index between 0.66 and 0.84). When *k* = 3, one cluster was highly stable and two stable; when *k* = 2, one cluster was highly stable and one stable (AJ values being 0.92 and 0.80). The clusters obtained for *k* = 2 and *k* = 3 are presented in Fig. 8. In both scenarios, the first cluster contains the series KF1 and KF2.

This result aligns with the values of the single pollution indices, which are as follows:

- The same classes with respect to ${I}_{\text{geo}}$ for all elements, except Pb and Zn (situated in neighboring classes).
- The same classes with respect to PI for all elements, except Cr (which falls within the range of unpolluted to low polluted sites).
- The same classes with respect to EF when computed based on Mn.
- The same neighboring classes with respect to EF when computed based on Fe.

**Fig. 9:**
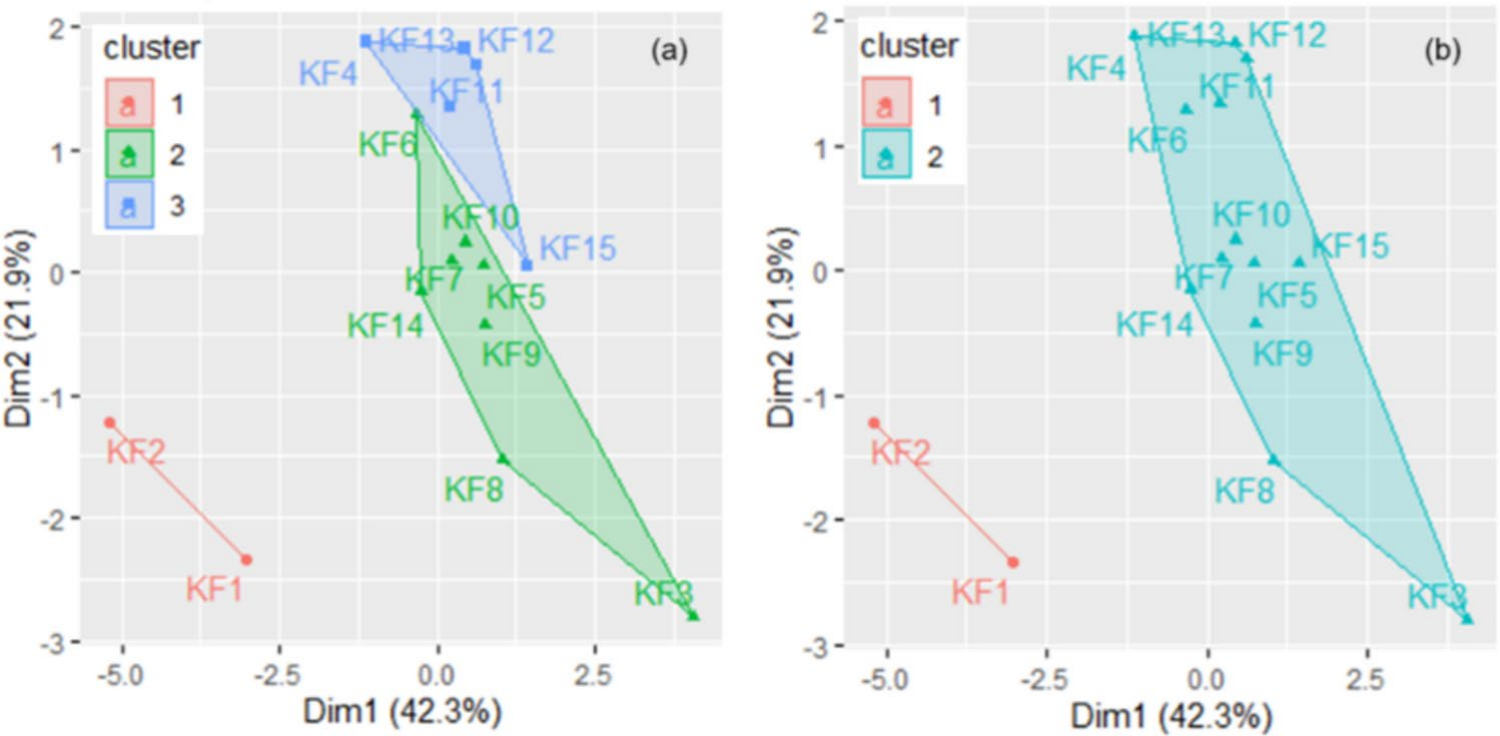
Clusters of the series in Khor Fakkan for **a** *k* = 3 and **b** *k* = 2

Similar observations apply to the third cluster in Fig. 9a.

**Fig. 10:**
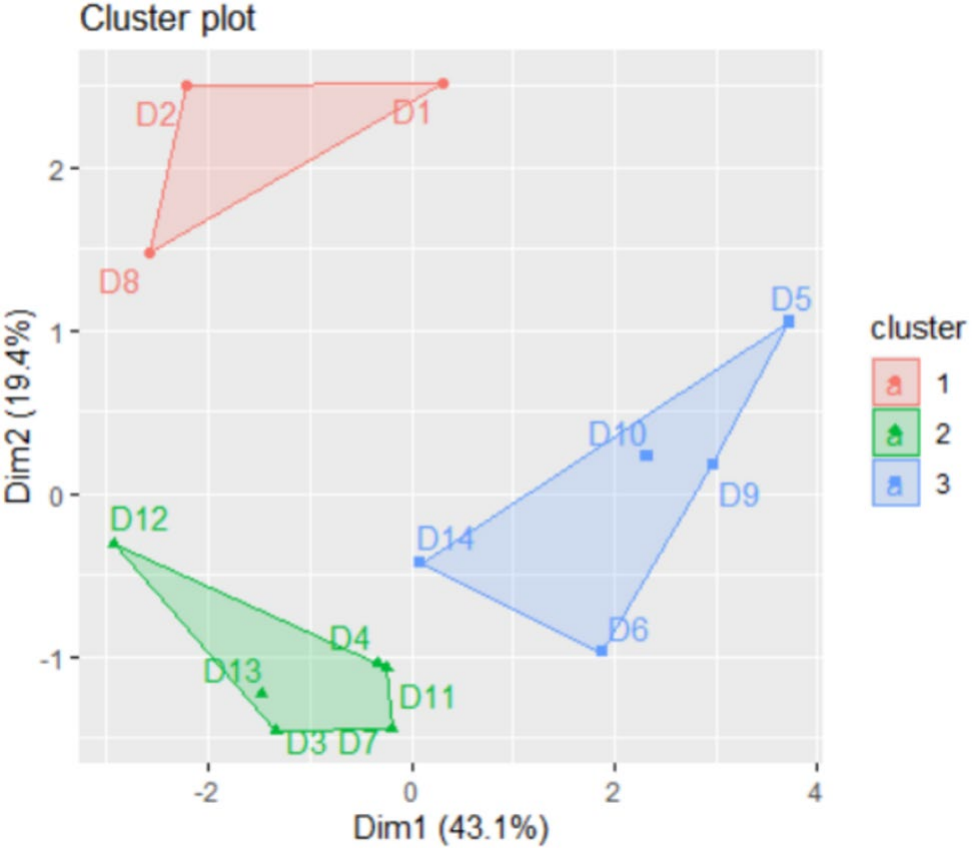
Clusters of the series in the Dubai region

For the series in the Dubai zone, analyzing the connectivity and stability across the possible scenario (*k* = 2, 3, or 6) revealed the following: the Connectivity and Dunn indexes suggested *k* = 2 as the optical choice, whereas the Silhouette index indicated *k* = 3. However, the highest AJ indices were observed when *k* = 3, leading to the selection of *k* = 3 for the series grouping. The clusters for the series in Dubai are shown in Fig. 10.

The series in the first cluster have the following characteristics. With respect to ${I}_{\text{geo}}$, they are classified into groups with similar contamination levels for Cd, Cr, Cu, Ba, Fe, and Mn. For Ni and Zn, they range from uncontaminated, or uncontaminated to moderately contamination. With respect to PI, they belong to the same contamination class for Cd, Cr, and Cu. Similar contamination with Pb and Co are observed in D1 and D2, while D8 shows lower pollution compared to D1 and D2. Additionally, D2 and D8 have smaller levels of pollution with Zn as compared to D1. Considering EF with respect to Mn, very high pollution levels with Zn are observed in D1 and D8. Considering the EF with respect to Fe, the enrichment levels for all elements are nearly similar. In the third cluster, we find sites with Zn belonging to various classes, Cd in unpolluted to low pollution classes, Cu, Ni, and Pb in classes varying from low to strong, and Zn mostly in the highest contamination class.

**Fig. 11:**
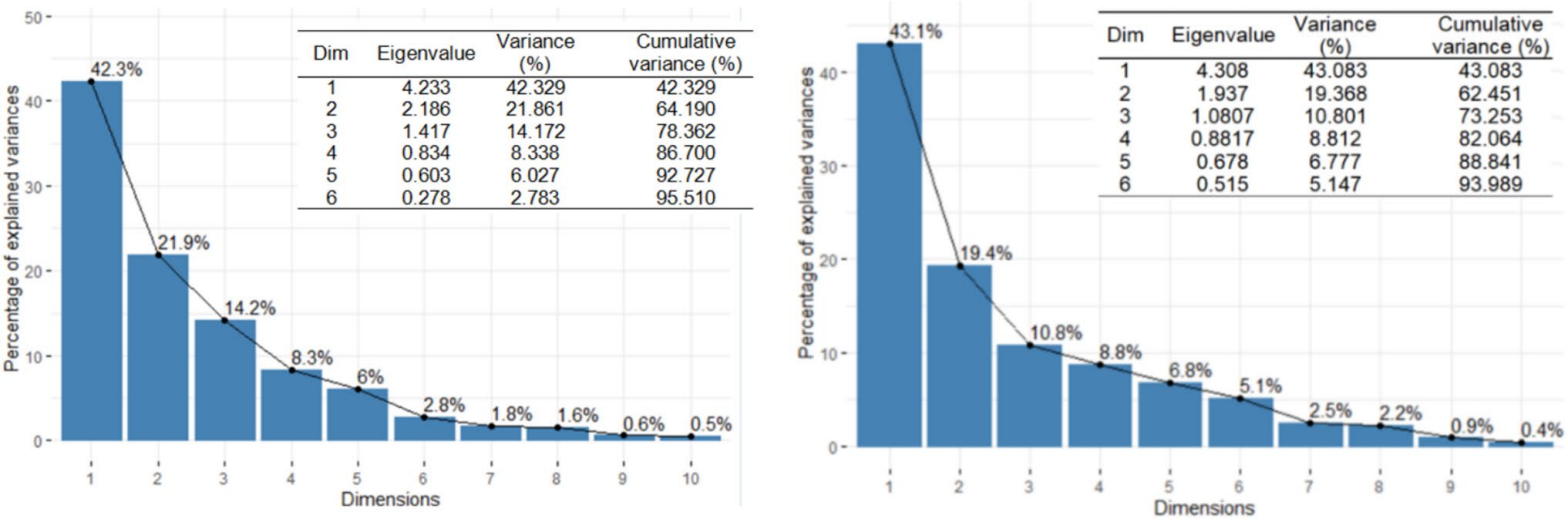
Clusters of the series in the (left) Khor Fakkan and (right) Dubai region

**Fig. 12:**
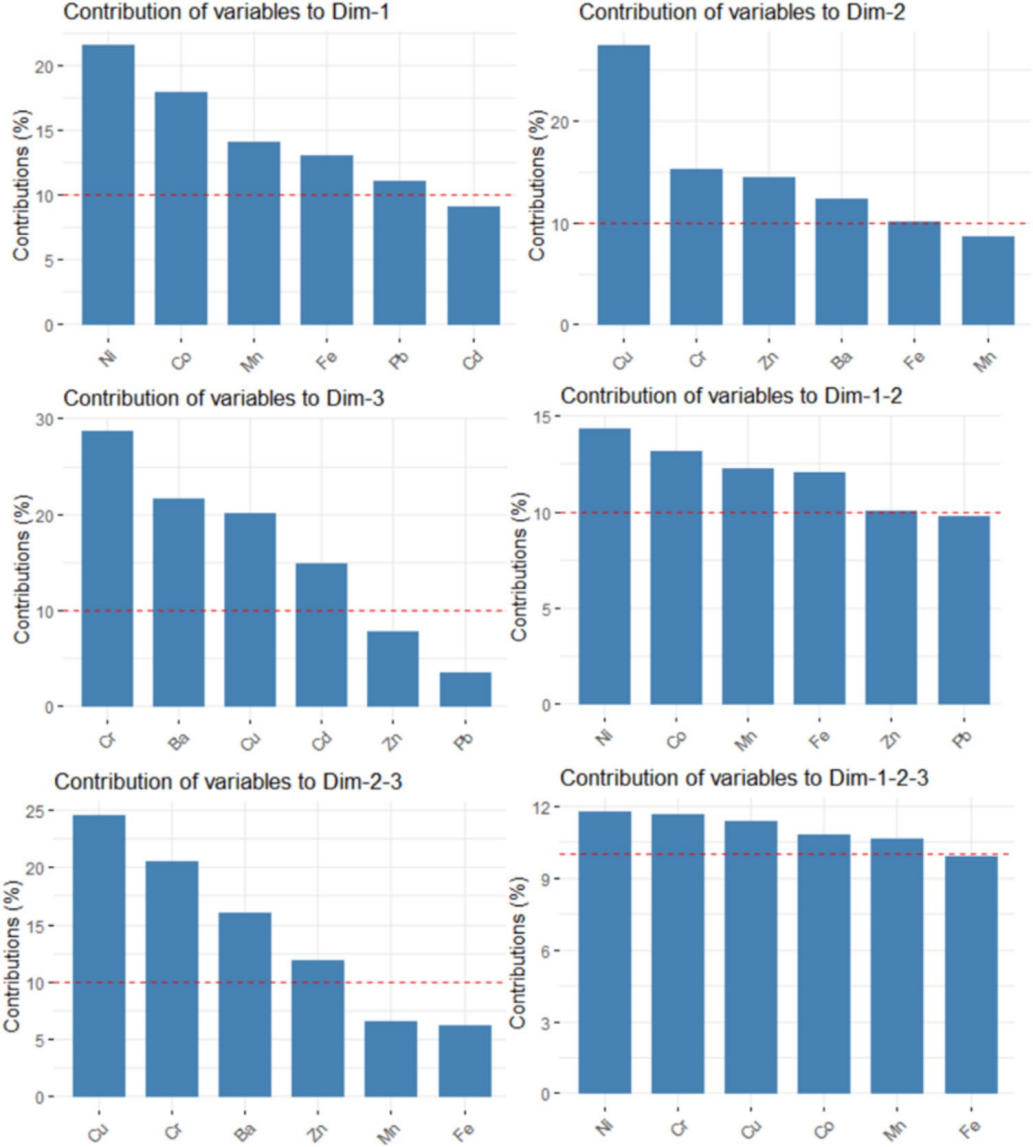
Contributions of the variables (elements) to the first three dimensions in Khor Fakkan. The read lines show the expected average contribution

To determine the main PCs, we computed the eigenvalues and the scree plot, which are presented in Fig. 11. In both cases, the first three eigenvalues are greater than 1, so we selected them as the main PCs. They explain about 78.362% and 73.253% of the total variance in the case of Khor Fakkan and Dubai, respectively. Adding the fourth component, 86.700% and 82.064% of the total variance is explained. Therefore, we selected the first three PCs as the main components for Khor Fakkan and four for Dubai. First, we present the results obtained for Khor Fakkan. The contributions of the elements on the first three dimensions (Dim) are presented in Fig. 12

Ni, Co, Mn, and Fe have the highest contributions to Dim-1, Cu, Cr, Zn, and Ba to Dim-2, Cr, Ba, Cu, and Cd to Dim-3. The most important contributions to the first two dimensions are those of Ni, Co, Mn, to Dim 2–3 are of Cu, Cr, Ba, and Zn, and to Dim-1–2-3 of Ni, Cr, Cu, and Co.

**Fig. 13:**
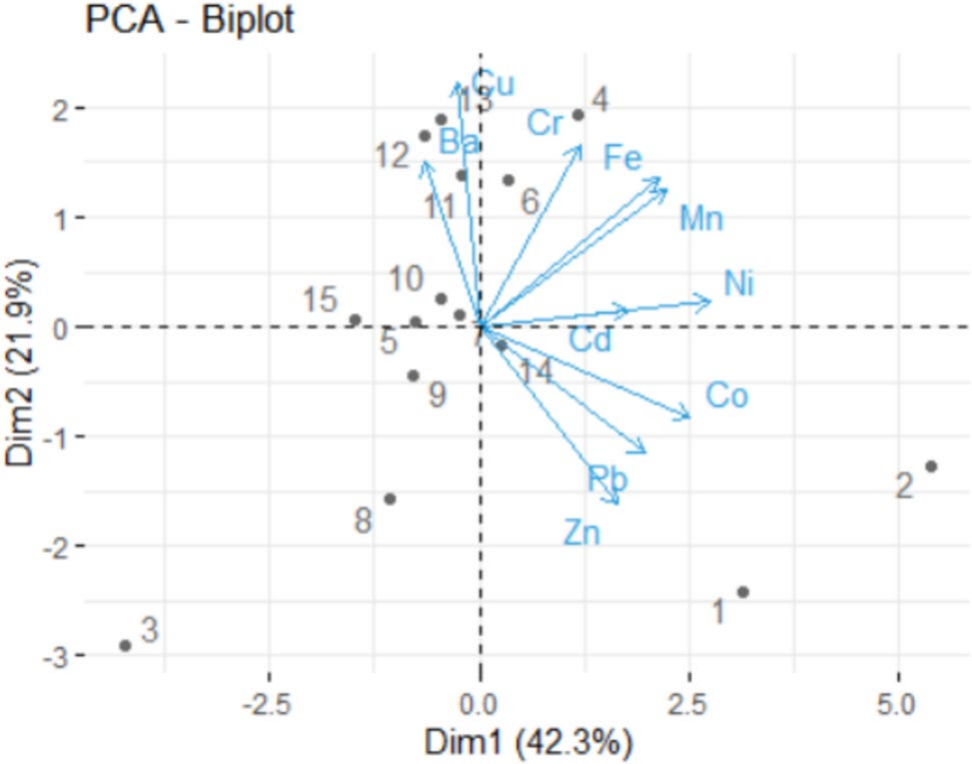
The biplot for Khor Fakkan

The PCA-biplot in Fig. 13 shows the contributions of the variables on the first two dimensions (represented with blue arrows) and those of the sites (denoted here by 1–15). The main contributions are those of the sites KF2, KF3, and KF1 on the Dim-1, and those of KF3, KF13, KF12, and KF8.

**Fig. 14:**
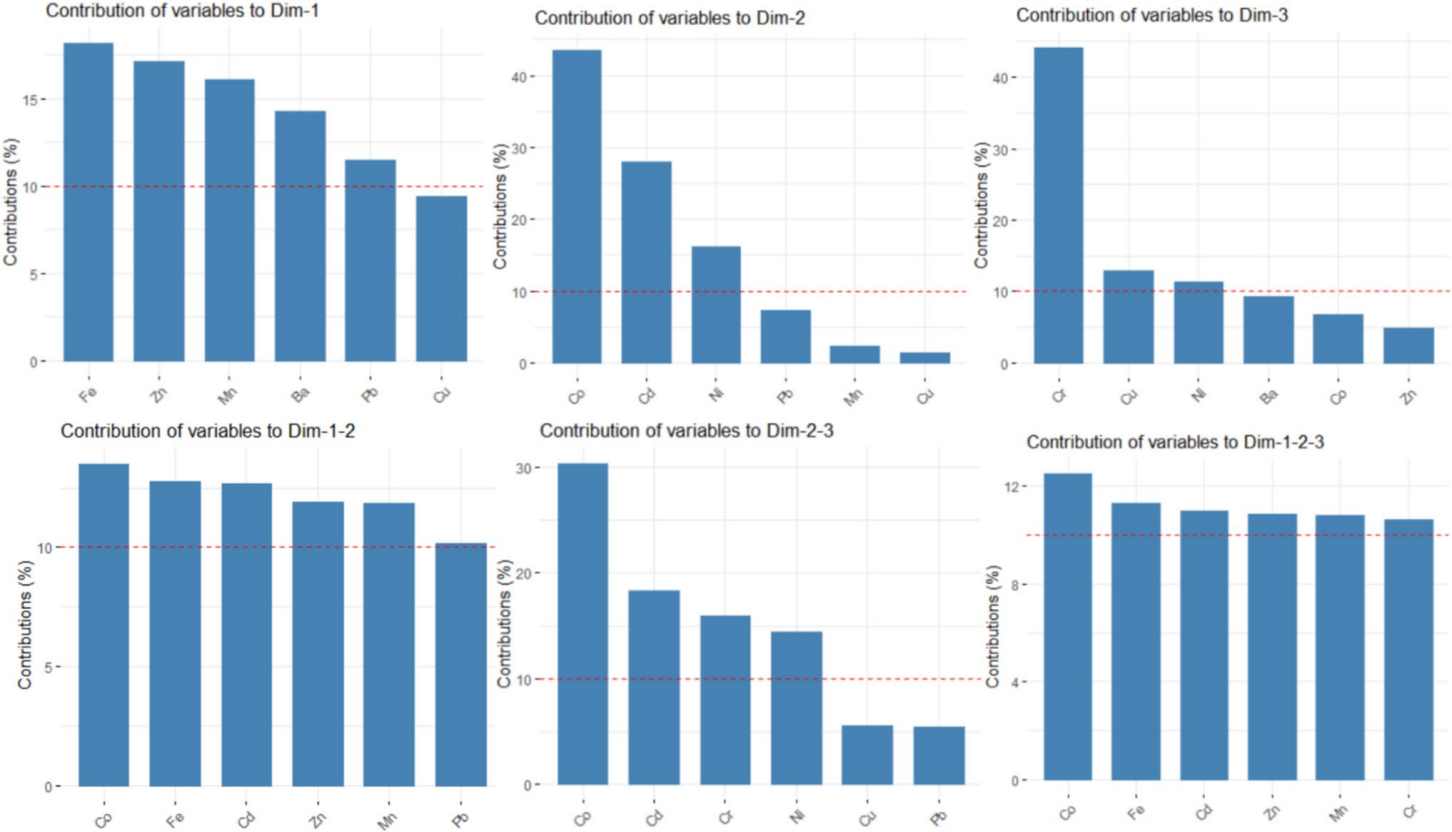
Contributions of the variables (elements) to the first three dimensions in Dubai

The contributions of the variables to the first three dimensions, to Dim-1–2, Dim-2–3, and Dim-1-2-3 in Dubai region are presented in Fig. 14. The highest contributions in Dim-1 are Fe, Zn, Mn, Ba, in Dim-2 are Co, Cd, and Ni, and in Dim-3 Cr, Cu, Ni.

**Fig. 15:**
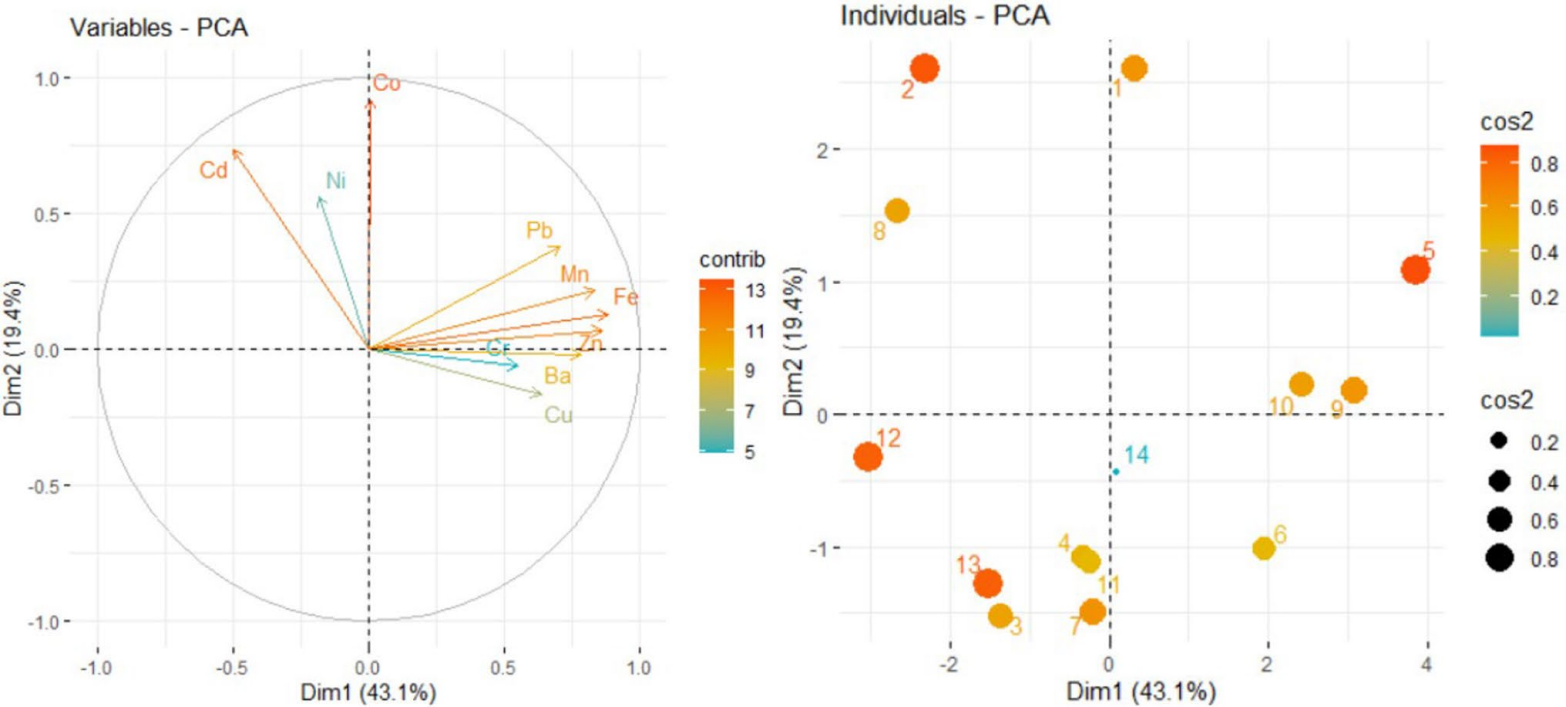
The variables-PCA (left) and Individuals-PCA (right)

Thus, the highest contributions to Dim-1–2 are Co, Fe, Cd, and Zn, and to Dim-2–3 are Co, Cd, Cr, and Ni, whereas to the first three dimensions are Co, Fe, Cd, and Zn. The variables-PCA (Fig. 15 (left)) shows the contributions of the variables on the first two PCs. It confirms the above findings. One may notice the negative contributions of Cd and Ni on PC1, and Cu and Ba on PC2.

The individuals—PCA is presented in Fig. 14(right), where cos2 is the quality of representation of the variables on factor map. High values of cos2 shows a good representation of the variable on the PCs. The highest contributions on the first component are those of the sites D5, D9 (on the positive direction of the horizontal axis), D12 (on the negative direction of the horizontal axis), whereas on the second axis are D2, D1, D8, and D7.

Comparing the results from the Dubai and Khor Fakkan regions we found that the main contributions of the first three PCs are not the same. For example, on PC1, Fe and Mn have contributions of the first or fourth order (as percentage), whereas and Mn has a third order contribution. The other two elements are different as contributions and percentages. On the second PC, Cu is the only element that has second order contributions on both regions, whereas, on PC3, Cr is situate on the first place. Overall, the highest contributions on the first three PCs are thise of Co, Fe, Cd, and Zn in Dubai, and Ni, Cr, Cu, Co, in Khor Fakkan.

The concentration of heavy metals in dust across Dubai and Khor Fakkan can be attributed to both lithological characteristics and industrial activities unique to each region. Dubai’s flat coastal plains, with aeolian deposits, act as natural reservoirs and dispersers of elements like lead (Pb) and cadmium (Cd) (Howari 2004). These heavy metals are further amplified by urban emissions, especially from vehicular traffic, construction activities, and port operations. In contrast, Khor Fakkan’s rugged terrain, formed from ultramafic rocks such as peridotites and gabbros of the semail ophiolite, naturally releases higher levels of nickel (Ni) and chromium (Cr) (Howari 2006). This geological foundation, coupled with industrial outputs from port activities and heavy traffic, significantly increases metal pollutants in dust, particularly Ni and Cr, as shown by elevated readings near sites like KF2, close to industrial zones.

## Conclusions

This study provides a comprehensive assessment of heavy metal contamination in urban road dust across two contrasting environments in the United Arab Emirates (Dubai and Khor Fakkan). Through a combination of geochemical indices, spatial analysis, multivariate statistics, and clustering methods, the research presents a novel approach to characterizing the extent, distribution, and potential sources of metal pollution in arid urban and semi-urban settings. The key contribution of this study lies in its comparative design, which distinguishes between pollution driven by natural lithological factors (ultramafic ophiolitic rocks in Khor Fakkan) and those resulting from intense anthropogenic pressures (vehicular emissions, industrial activities, and urban sprawl in Dubai). Notably, the high levels of Ni and Cr in Khor Fakkan are associated with its geological makeup, whereas Cd, Zn, and Pb are more enriched in Dubai and are largely attributed to anthropogenic inputs. This differentiation is crucial for developing targeted environmental management strategies.

The integration of pollution indices with clustering and principal component analyses provides a robust diagnostic framework to evaluate urban environmental risks, an approach not commonly applied in arid regions. Importantly, the study introduces a localized pollution representation tool (EF_local) that enhances the interpretation of contamination data in densely sampled zones and improves decision-making at the neighborhood scale. Moreover, the findings establish region-specific baselines for metal contamination in road dust, which are vital for long-term monitoring and health risk assessments. By focusing on an arid region undergoing rapid urbanization, this study fills a significant geographic and thematic gap in the road dust literature. It offers valuable insights into how geological and anthropogenic drivers intersect in shaping dust pollution patterns, contributing to global understanding of environmental contamination in dryland urban systems.

However, an important consideration in interpreting spatial variations in road dust contamination is the differential implementation of dust suppression measures across study locations. While both Dubai and Khor Fakkan experience heavy vehicular and industrial activities, the frequency and extent of dust mitigation practices—including street cleaning, water spraying, and chemical dust suppressants—are not uniformly documented. Limited anecdotal evidence suggests that Dubai implements more frequent municipal street sweeping and and water spraying, particularly in high-traffic urban zones, while such practices appear less routine in Khor Fakkan’s mountainous and industrial districts. This inconsistency in dust mitigation practices represents a potential confounding variable when interpreting contamination patterns. Future studies should systematically assess dust suppression practices to better correlate pollution levels with policy effectiveness and establish more robust comparisons between study areas. Future studies should incorporate systematic assessments of dust suppression practices alongside source apportionment and health risk modeling to improve pollutant attribution and support the development of targeted environmental and public health policies in similar arid environments worldwide.

## Data Availability

The raw data supporting the conclusions of this article will be made available by the authors, without undue reservation.

## References

[CR1] Abbasi S, Keshavarzi B, Moore F, Delshab H, Soltani N, Sorooshian A (2017) Investigation of microrubbers, microplastics and heavy metals in street dust: a study in Bushehr city, Iran. Environ Earth Sci 76:1–9

[CR2] Al-Taani AA, Nazzal Y, Howari FM, Yousef A (2019a) Long-term trends in ambient fine particulate matter from 1980 to 2016 in United Arab Emirates. Environ Monit Assess 191:1–19 10.1007/s10661-019-7259-9 30734105

[CR3] Al-Taani AA, Nazzal Y, Howari FM (2019b) Assessment of heavy metals in roadside dust along the Abu Dhabi–Al Ain National Highway, UAE. Environ Earth Sci 78:1–13

[CR4] Al-Taani AA, Nazzal Y, Howari FM, Iqbal J, Orm NB, Xavier CM, Bărbulescu A, Sharma M, Dumitriu CS (2021) Contamination assessment of heavy metals in agricultural soil, in the Liwa area (UAE). Toxics 9(3):53 33801890 10.3390/toxics9030053 PMC8000652

[CR5] Al-Taani AA, Nazzal Y, Howari FM, Iqbal J, Naseem M, Sharma M, Xavier CM, Papandreou D, Maloukh L, Ambika L, Salem IB, Bsoul AA, Farok HM (2023) Metal composition and contamination assessment of urban roadway dusts on the Abu dhabi-Liwa Highway, UAE. Front Environ Sci 11:1157101

[CR6] Amato F, Querol X, Johansson C, Nagl C, Alastuey A (2010) A review on the effectiveness of street sweeping, washing and dust suppressants as urban PM control methods. Sci Total Environ 408(16):3070–3084 20488509 10.1016/j.scitotenv.2010.04.025

[CR7] Arslan H (2001) Heavy metals in street dust in Bursa, Turkey. J Trace Microprobe Tech 19(3):439–445

[CR8] Bărbulescu A (2016) A new method for estimation the regional precipitation. Water Resour Manag 30(1):33–42

[CR9] Batayneh A, Elawadi E, Zaman H, Al-Taani AA, Nazzal Y, Ghrefat H (2014) Environmental assessment of the Gulf of Aqaba coastal surface waters, Saudi Arabia. J Coast Res 30(2):283–290

[CR10] Bilos C, Colombo JC, Skorupka CN, Presa MR (2001) Sources, distribution and variability of airborne trace metals in La Plata City area, Argentina. Environ Pollut 111(1):149–158 11202709 10.1016/s0269-7491(99)00328-0

[CR11] Brock G, Pihur V, Datta S, Datta S (2008) Clvalid: an R package for cluster validation. J Stat Softw 25:1–22. 10.18637/jss.v025.i04

[CR12] Brown JH, Cook KM, Ney FG, Hatch T (1950) Influence of particle size upon the retention of particulate matter in the human lung. Am J Public Health Nations Health 40(4):450–480 18017198 10.2105/ajph.40.4.450 PMC1528473

[CR13] Cai K, Li C (2019) Street dust heavy metal pollution source apportionment and sustainable management in a typical city—Shijiazhuang, China. Int J Environ Res Public Health 16(14):2625 31340519 10.3390/ijerph16142625 PMC6678876

[CR14] Cattell RB (1966) The scree test for the number of factors. Multivar Behav Res 1(2):245–276 10.1207/s15327906mbr0102_10 26828106

[CR15] Charrad M, Ghazzali N, Boiteau V, Niknafs A. (2022) Package NbClust. Determining the Best Number of Clusters in a Data Set. https://cran.r-project.org/web/packages/NbClust/ NbClust.pdf

[CR16] Coman G, Draghici C. (2011) Heavy metals activity mechanisms at cellular level and possible action on children’s bodies. In: Simeonov, L., Kochubovski, M., Simeonova, B. (eds), Environmental Heavy metal pollution and effects on child mental development. NATO Science for peace and security series C: environmental security, vol 1.; Springer, Dordrecht, Netherlands, pp. 145–158

[CR17] Du Y, Gao B, Zhou H, Ju X, Hao H, Yin S (2013) Health risk assessment of heavy metals in road dusts in urban parks of Beijing, China. Proc Environ Sci 18:299–309

[CR18] RTA Dubai (2020) Annual report. Dubai Roads and Transport Authority

[CR19] Eqani SAMAS, Kanwal A, Bhowmik AK, Sohail M, Ullah R, Ali SM, Alamdar A, Ali N, Fasola M, Shen H (2016) Spatial distribution of dust–bound trace elements in Pakistan and their implications for human exposure. Environ Pollut 213:213–222 26901073 10.1016/j.envpol.2016.02.017

[CR20] Faisal M, Wu Z, Wang H, Hussain Z, Azam MI (2021) Human health risk assessment of heavy metals in the urban road dust of Zhengzhou metropolis, China. Atmosphere 12(9):1213

[CR21] Gong Q, Deng J, Xiang Y, Wang Q, Yang L (2008) Calculating pollution indices by heavy metals in ecological geochemistry assessment and a case study in parks of Beijing. J China Univ Geosci 19:230–241

[CR22] Grigoratos T, Martini G (2015) Brake wear particle emissions: a review (2015). Environ Sci Pollut Res 22:2491–2504 10.1007/s11356-014-3696-8 PMC4315878 25318420

[CR23] Habib RZ, Al Kendi R, Ghebremedhin F, Elkashlan M, Iftikhar SH, Poulose V, Ramachandran T, Mourad AH, Hamed F, Thiemann T (2022) Tire and rubber particles in the environment—a case study from a hot arid region. Front Environ Sci 10(10):1009802

[CR24] Håkanson L (1980) An ecological risk index for aquatic pollution control—a sedimentological approach. Water Res 14(8):975–1001

[CR25] Hennig C (2019) Cluster validation by measurement of clustering characteristics relevant to the user. In: Skiadas CH (ed) Data analysis and applications 1: clustering and regression, modeling estimating, forecasting and data mining, vol 2. Wiley, New York, pp 1–24

[CR26] Hennig C. (2020) fpc: Flexible procedures for clustering. R package version 2.2–9, https://CRAN.R-project.org/package=fpc

[CR27] Howari FM (2004) Heavy metal speciation and mobility assessment in arid soils of Dubai (United Arab Emirates). Environ Monit Assess 35(2):123–145

[CR29] Howari FM (2006) Heavy metal speciation and mobility assessment of arid soils in the vicinity of Al Ain Landfill, United Arab Emirates. Int J Environ Pollut 22(6):721–731

[CR30] Inengite AK, Abasi CY, Walter C (2015) Application of pollution indices for the assessment of heavy metal pollution in flood impacted soil. Int Res J Pure Appl Chem 8:175–189

[CR31] Inyang HI, Bae S (2006) Impacts of dust on environmental systems and human health. J Hazard Mater 132(1):5–6 10.1016/j.jhazmat.2005.11.082 16442715

[CR32] Jolliffe I. (2014) Principal component analysis; Wiley Stats. Ref. Online: Hoboken, NJ, USA

[CR33] Joun H, Kokkalas S, NTombros S (2019) Recycled oceanic crust as a source for tonalite intrusions in the mantle section of the Khor Fakkan block, Semail ophiolite (UAE). Geosci Front 10(3):1187–1210

[CR34] Kaiser HF (1960) The application of electronic computers to factor analysis. Educ Psychol Meas 20:141–151

[CR35] Kaufmann L, Rousseeuw P. (1986) Clustering by means of Medoids. https://www.researchgate.net/publication/243777819_Clustering_by_Means_of_Meds#fullTextFileContent

[CR36] Khodadadi N, Amini A, Dehbandi R (2022) Contamination, probabilistic health risk assessment and quantitative source apportionment of potentially toxic metals (PTMs) in street dust of a highly developed city in north of Iran. Environ Res 210:112962 35182599 10.1016/j.envres.2022.112962

[CR37] Kowalska JB, Mazurek R, Gąsiorek M, Zaleski T (2018) Pollution indices as useful tools for the comprehensive evaluation of the degree of soil contamination—a review. Environ Geochem Health 40:2395–2420. 10.1007/s10653-018-0106-z 29623514 10.1007/s10653-018-0106-z PMC6280880

[CR38] Lindsay WL (1979) Chemical equilibrium in soils. John Wiley & Sons, New York

[CR39] Lu X, Wang L, Lei K, Huang J, Zhai Y (2009) Contamination assessment of copper, lead, zinc, manganese and nickel in street dust of Baoji, NW China. J Hazard Mater 161(2–3):1058–1062 18502044 10.1016/j.jhazmat.2008.04.052

[CR40] Maloukh L, Nazzal Y, Kumarappan A, Howari FM, Ambika LK, Yahmadi R, Sharma M, Iqbal J, Al-Taani AA, Salem IB, Xavier CM, Naseem M (2023) Metagenomic analysis of the outdoor dust microbiomes: a case study from Abu Dhabi, UAE. Atmosphere 14(2):327

[CR41] Manno E, Varrica D, Dongarrà G (2006) Metal distribution in road dust samples collected in an urban area close to a petrochemical plant at Gela, Sicily. Atmos Environ 40(30):5929–5941

[CR42] Mayer PM, Moran KD, Miller EL, Brander SM, Harper S, Garcia-Jaramillo M, Carrasco-Navarro V, Ho KT, Burgess RM, Hampton LM, Granek EF (2024) Where the rubber meets the road: emerging environmental impacts of tire wear particles and their chemical cocktails. Sci Total Environ 7:171153 10.1016/j.scitotenv.2024.171153 PMC11214769 38460683

[CR43] Moreno T, Karanasiou A, Amato F, Lucarelli F, Nava S, Calzolai G, Chiari M, Coz E, Artíñano B, Lumbreras J, Borge R, Boldo E, Linares C, Alastuey A, Querol X, Gibbons W (2013) Daily and hourly sourcing of metallic and mineral dust in urban air contaminated by traffic and coal-burning emissions. Atmos Environ 68:33–44

[CR44] Müller G (1969) Index of geoaccumulation in sediments of the Rhine River. GeoJournal 2:108–118

[CR45] National Centre of Meteorology-UAE https://www.ncm.gov.ae/

[CR46] Nazzal Y, Bărbulescu A, Howari FM, Al-Taani AA, Iqbal J, Xavier CM, Sharma M, Dumitriu CȘ (2021a) Assessment of metals concentrations in soils of Abu Dhabi Emirate using pollution indices and multivariate statistics. Toxics 9(5):95 33923007 10.3390/toxics9050095 PMC8146448

[CR47] Nazzal Y, Orm NB, Barbulescu A, Howari FM, Sharma M, Badawi AE, Al-Taani AA, Iqbal J, Ktaibi FE, Xavier CM, Dumitriu CS (2021b) Study of atmospheric pollution and health risk assessment: a case study for the Sharjah and Ajman Emirates (UAE). Atmosphere 12(11):1442

[CR48] Nazzal Y, Bărbulescu A, Sharma M, Howari F, Naseem M (2023) Evaluating the contamination by indoor dust in Dubai. Toxics 11(11):933 37999585 10.3390/toxics11110933 PMC10674184

[CR49] Panko JM, Chu J, Kreider ML, Unice KM (2013) Measurement of airborne concentrations of tire and road wear particles in urban and rural areas of France, Japan, and the United States. Atmos Environ 1(72):192–199

[CR50] Roy S, Gupta SK, Prakash J, Habib G, Kumar P (2022) A global perspective of the current state of heavy metal contamination in road dust. Environ Sci Pollut Res Int 29:33230–33251 35022986 10.1007/s11356-022-18583-7

[CR51] Semerjian L, Adeniji AO, Shanableh A, Semreen MH, Mousa M, Abass K, Okoh A (2024) Assessment of elemental chemistry, spatial distribution, and potential risks of road-deposited dusts in Sharjah, United Arab Emirates. Heliyon 10(7):e29088 38617947 10.1016/j.heliyon.2024.e29088 PMC11015408

[CR52] Sutherland RA (2000) Bed sediment-associated trace metals in an urban stream, Oahu, Hawaii. Environ Geol 39(6):611–627

[CR53] Tchounwou PB, Yedjou CG, Patlolla AK, Sutton DJ. (2012) Heavy metal toxicity and the environment. In: Luch, A. (eds) Molecular, clinical and environmental toxicology. Experientia supplementum, vol. 101, Springer, Basel, pp. 133–164 10.1007/978-3-7643-8340-4_6 PMC4144270 22945569

[CR54] Thorpe A, Harrison RM (2008) Sources and properties of non-exhaust particulate matter from road traffic: a review. Sci Total Environ 400(1–3):270–282 18635248 10.1016/j.scitotenv.2008.06.007

[CR55] Tomlinson DL, Wilson JG, Harris CR, Jeffrey DW (1980) Problems in the assessment of heavy-metal levels in estuaries and the formation of a pollution index. Helgol Meeresunter 33:566–575

[CR56] U.S. Environmental Protection Agency (USEPA) (1996) Method 3050B: Acid digestion of sediments, sludges, and soils. National center for environmental assessment, Washington, DC. https://www.epa.gov/sites/default/files/2015-06/documents/epa-3050b.pdf

[CR57] Wang G, Fowler BA (2008) Roles of biomarkers in evaluating interactions among mixtures of lead, cadmium and arsenic. Toxicol Appl Pharmacol 233(1):92–99 18325558 10.1016/j.taap.2008.01.017

[CR58] Willers S, Gerhardsson L, Lundh T (2005) Environmental tobacco smoke (ETS) exposure in children with asthma—relation between lead and cadmium, and cotinine concentrations in urine. Respir Med 99(12):1521–1527 16291074 10.1016/j.rmed.2005.03.017

[CR59] Yongming H, Peixuan D, Junji C, Posmentier ES (2006) Multivariate analysis of heavy metal contamination in urban dusts of Xi’an, Central China. Sci Total Environ 355(1–3):176–186 15885748 10.1016/j.scitotenv.2005.02.026

[CR60] Zhang C, Qiao Q, Appel E, Huang B (2012) Discriminating sources of anthropogenic heavy metals in urban street dusts using magnetic and chemical methods. J Geochem Explor 119:60–75

